# Identification of patient-specific and tumor-shared T cell receptor sequences in renal cell carcinoma patients

**DOI:** 10.18632/oncotarget.15064

**Published:** 2017-02-03

**Authors:** Chiara Massa, Harlan Robins, Cindy Desmarais, Dagmar Riemann, Corinna Fahldieck, Paolo Fornara, Barbara Seliger

**Affiliations:** ^1^ Institute of Medical Immunology, Martin Luther University Halle-Wittenberg, Halle, Saale 06112, Germany; ^2^ Adaptive Biotechnologies Corp, Seattle, WA 98102, USA; ^3^ Clinic of Urology, Martin Luther University Halle-Wittenberg, Halle, Saale 06112, Germany

**Keywords:** TCR sequencing, renal cell carcinoma, immune response, immune monitoring, immune therapy

## Abstract

A major requirement for cancer immunotherapy is the development of biomarkers for prognosis and for monitoring therapy response. In an attempt to evaluate the immune response of renal cell carcinoma (RCC) patients, tumor lesions and / or blood samples from 12 RCC patients underwent deep T cell receptor (TCR) sequencing. Despite the low number of samples, different TCR distribution patterns could be detected. Most of the RCC patients presented “patient-specific” TCR sequences, and those clonotypes were present at higher frequency in tumor lesions suggesting a specific extravasation from the blood. Comparison among the tumor samples revealed also “patient-shared” TCR patterns. Indeed, a central core of 16 different TCRs were shared by 3 patients, whereas other 6 patients shared between 4 and 6 TCR sequences, with two sub-groups sharing 12 to 17 different clonotypes. The relative frequencies of shared clonotypes were very different varying from < 1% to a maximum of 37% of the total TCR repertoire. These data confirm the presence of tumor-specific TCR within the cancer tissue and suggest the existence of shared epitopes among different patients that might be used as targets for tumor immunotherapy.

## INTRODUCTION

Renal cell carcinoma (RCC) occurs with an incidence of 2-3% and is the 10^th^ most common cancer type [1, 2]. While the 5-year survival rate for localized RCC is above 70%, it drops to 12% when the disease has spread to distant organs, which is the case in more than half of the patients at presentation [2]. In the search for alternative or complementation therapies to surgical eradication for patients with advanced disease, the introduction of novel targeted agents has resulted in a better management of metastatic RCC [3, 4]. Although the therapeutic options targeting the VHL/HIF pathway with different tyrosine kinase inhibitors did improve the overall and progression-free survival, durable complete responses remain elusive [5, 6]. In addition, the high immunogenicity of RCC has led to the implementation of various immunotherapeutic approaches. In the setting of adjuvant immunotherapy, the long-term use of the cytokines interleukin (IL)-2 [7] and IFNα [8] has been extended to IL-21 [9], while in parallel new immune modulators have been developed, such as check point inhibitors acting on the PD1/PDL1 axis [10]. In the setting of active immunotherapy patients have been vaccinated with “undefined” tumor specificities using autologous or allogeneic tumor cells whose immunogenicity has been enhanced by transfection of immune stimulatory molecules [11–13], IFNγ treatment [14] or by loading their lysate / fusing them with dendritic cells (DC) [15–17]. With the identification of antigenic epitopes expressed by RCC [18–22], various clinical trials have evaluated their functionality [23, 24]. In addition, adoptive immunotherapy of more or less antigen-specific strategies has been implemented. Next to cytokine induced killer cells obtained from the peripheral blood [25, 26], whose antigen specificity is now boosted by co-culture with tumor-lysate pulsed autologous DC [27], tumor infiltrating lymphocytes (TIL) also represent a valuable source of tumor-antigen reactive effector cells [28]. More recently, the cloning of T cell receptor (TCR) specific for various tumor antigens and the development of chimeric antigen receptors (CARs) [29] have introduced a new level of antigen specificity to adoptive therapy by the use of engineered T cells. RCC clinical trials using CARs based on the G250 antibody recognizing carbonic anhydrase IX [30, 31] and TCRs against common tumor antigens like p53, MAGE-A3 and NY-ESO-1 are currently ongoing (as stated on theclinicaltrials.gov web site). In order to expand the repertoire of TCRs to be used in adoptive therapy against RCC, many groups have undergone the cumbersome protocol of expanding and sub-cloning tumor reactive T cells from the TIL of RCC patients in order to isolate the respective TCR [32–34]. An alternative approach can be the usage of high throughput sequencing applied to immunologic molecules, such as the TCR, but also immunoglobulin. By combining a step of amplification with primers targeting the constant region upstream and downstream the Variability (V) / Join (J) / Diversity (D) alleles of the TCR with the sequencing of all amplified products, deep TCR sequencing allows the complete characterization of the whole TCR repertoire present in the analyzed sample. This technique has already been used to follow minimal residual disease in T and B cell leukemia, where the specific TCR / immunoglobulin of the leukemic cells can be detected with much higher sensitivity than with previous techniques, but can also be used to characterize the whole repertoire of specificities in setting of infection or cancer, thus providing a database to search for possible therapeutic useful TCRs.

In order to deepen our knowledge of the TCR repertoire against RCC samples from twelve RCC patients that underwent surgical resection of the primary tumor were analyzed by deep TCR sequencing. Comparison of the frequency of the TCR sequences between the blood and tumor infiltrate of the various patients identified a high number of tumor-enriched sequences. Among them we found not only patient-specific sequences, that could target single tumor-restricted mutations important in the setting of personalized therapy, but also various TCR that were shared by more patients and that might target widespread expressed RCC-associated tumor antigens.

## RESULTS AND DISCUSSION

### Efficiency of the process

In this study, the TCR repertoire of tumor infiltrating and circulating lymphocytes was evaluated in 12 RCC patients that underwent surgical removal of the primary tumor in the Urology Department of the University Clinic of the Martin Luther University. In some cases blood samples were also collected at the time of surgery and peripheral blood mononuclear cells (PBMC) were isolated. To compare different starting material both fresh-frozen and paraffin-embedded tumor tissue were evaluated and DNA was extracted without previous purification of the lymphocytes. Patients’ characteristics and derived samples are provided in Supplementary Table 1.

TCR sequencing was successful on all sample types, with the highest success rate on PBMC samples (seven out of eight, 88% successfully sequenced) and the worst on paraffin-embedded tissues (six out of ten, 60% success) with fresh-frozen probes having intermediate results (nine out of twelve, 75% success). In light of the fact that the samples were analyzed more than 7 years after surgery, these success rates indicate that it is possible to obtain suitable data from older samples, e.g. paraffin-embedded tissues from the surgery of the primary tumor that were not completely used up during diagnostic evaluation.

**Figure 1:**
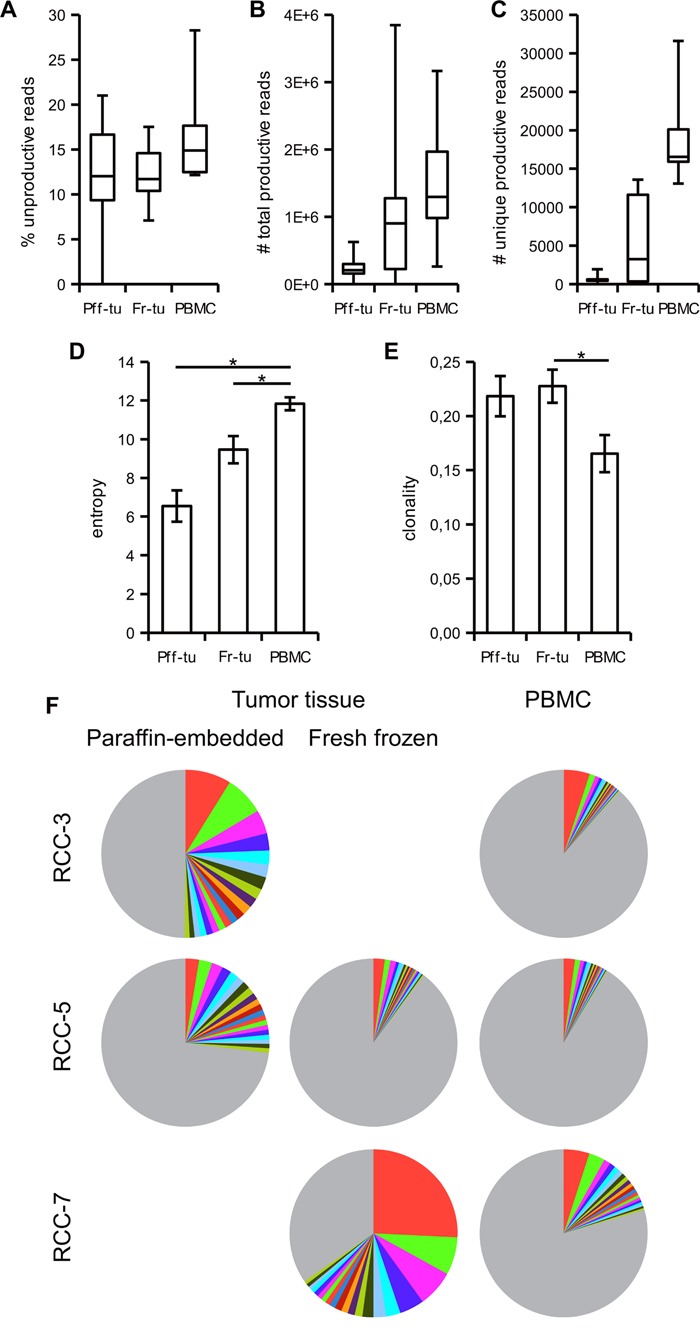
Tumor infiltrating lymphocytes have a more restricted TCR repertoire than blood resident T cells Paraffin-embedded (Pff-tu) and fresh-frozen (Fr-tu) tumor tissue from RCC patients underwent deep TCR sequencing together with the respective PBMC sample. Frequency of unproductive sequences out of frame or with stop codon; **A**., total number of productive **B**. and of unique reads **C**. are shown as a box-and-whiskers plot, displaying median, first and third quartile together with minimum and maximum values. The entropy D. and clonality E. of the samples were calculated as described in materials and methods and shown as mean ± SE. * p < 0,05 in one-way ANOVA for independent samples. F. Frequency of the top 20 TCR sequences from three different patients in the various specimens is shown as pie chart. The colors are automatically given by the software and do not correspond to identical TCR clonotypes. Grey represents the sum of the remaining TCRs.

Regarding the output, 13,67 ± 1,18% (range from 0 to 28,26%) of the total reads in the various samples were unproductive, i.e. out of frame or containing a stop codon. Comparison among the different sample types did not highlight any clear differences (Figure 1A), suggesting that the preparation or preservation technique does not influence the integrity of the template nor is adding artefacts resulting in enhanced levels of unproductive reads. Since no link was found with the total number of reads (data not shown), the unproductive reads were also not related to technical problems of the sequencing protocol. All following statistical evaluations and frequency calculations excluded those sequences and are only based on the productive reads, i.e. those with an in frame sequence able to be translated into a functional TCR. PBMC samples provided the highest total as well as unique sequencing read counts (Figure 1B-1C), while the fresh-frozen samples provided consistently more results than paraffin-embedded tumor tissues. The diversity of reads present in the different samples determined via the Shannon Entropy statistic highlighted a statistically significant higher diversity of the PBMC over tumor samples (Figure 1D) and reciprocally a higher clonality in the tumor infiltrate (Figure 1E). As a further index of the more restricted number of clonotypes representing the TCR repertoire of the tumor infiltrate, only a mean of 2,7 TCR sequences (range 1 to 5) had a frequency above 1% in the PBMC, whereas in the paraffin-embedded and fresh-frozen tumor tissues 13,5 and 12,4 TCR sequences were present, respectively (range 8 to 24 and 3 to 32, respectively; Table 1). Moreover, evaluation of the top 20 clones in each sample resulted in a coverage of the total measured TCR repertoire of 45,1% ± 9,9 and 29,4% ± 7,0 in the paraffin-embedded and the fresh-frozen tumor tissue, respectively, but only 11,9% ± 1,7 of the total TCR repertoire in PBMC (Table 1). Representative data of the TCR repertoire coverage from the top 20 clones from three patients are shown in Figure 1F as pie chart.

**Table 1:** Summary data of the TCR sequences in the various samples

Patient ID	# sequences with frequency > 1%			Top 20 clones’ added frequency		
	Pff-tu^a^	Fr-Tu^b^	PBMC	Pff-tu^a^	Fr-Tu^b^	PBMC
RCC-1		21			55,1	
RCC-2	24		1	38,3		9,1
RCC-3	18		2	50,1		11,5
RCC-4	9		3	26,1		9,7
RCC-5	12	3	2	27	10,2	8,6
RCC-6		3	1		15,1	8,8
RCC-7		13	5		65,3	20,5
RCC-8		3	5		12,3	15,3
RCC-9		11			28	
RCC-10	8	32		91,2	14	
RCC-14	10	20		38	47	
RCC-16		6			17,9	
Mean ± SE	13,5 ± 2,6	12,4 ± 3,4	2,7 ± 0,6	45,1 ± 9,9	29,4 ± 7	11,9 ± 1,7

^a^: Pff-tu: paraffin-embedded tumor tissue; ^b^: Fresh-frozen tumor tissue

### Within-patient comparison

Due to unsuccessful sequencing, all three sample types could be sequenced and compared to only for one patient (RCC-5). For the other donors there are a total of six cases, in which the repertoire of circulating lymphocytes can be compared to that of TIL, with three derived from paraffin-embedded and three from fresh-frozen tumor tissues (see Supplementary Table 1). In addition there are also two additional donors (RCC-10 and RCC-14), in which a comparison between the TCR repertoire found in fresh-frozen and paraffin-embedded tumor tissues can be performed.

#### Spatial distribution of T cells in tumor samples

For three patients it was possible to compare results between paraffin-embedded and fresh-frozen tumor tissues not only allowing the comparison of the two preservation techniques for the sequencing procedures, but also spatial comparison within the same tumor. While previous reports of TCR sequencing have highlighted a significant degree of homogeneity in ovarian cancer, more differences were found in RCC tumors [35, 36].

Regarding the efficacy of the process a more than hundred fold higher number of total reads (data not shown) and unique clonotypes were obtained from the fresh-frozen tissues in two patients, whereas in the remaining patient the paraffin-embedded sample provided a slightly higher output than the fresh-frozen tumor (Table 2). These data, together with the higher success rate, would suggest a better performance of fresh-frozen tumor tissues for TCR sequencing although paraffin-embedded tissues can still be used.

**Table 2:** Number of unique reads and shared clonotypes in the various tumor samples from the RCC patients indicated

Patient ID	Pff-tu^a^	Fr-Tu^b^	shared
RCC-5	565	11064	262
RCC-10	9	11494	4
RCC-14	545	339	75

^a^: Pff-tu: paraffin-embedded tumor tissue; ^b^: Fresh-frozen tumor tissue

**Figure 2:**
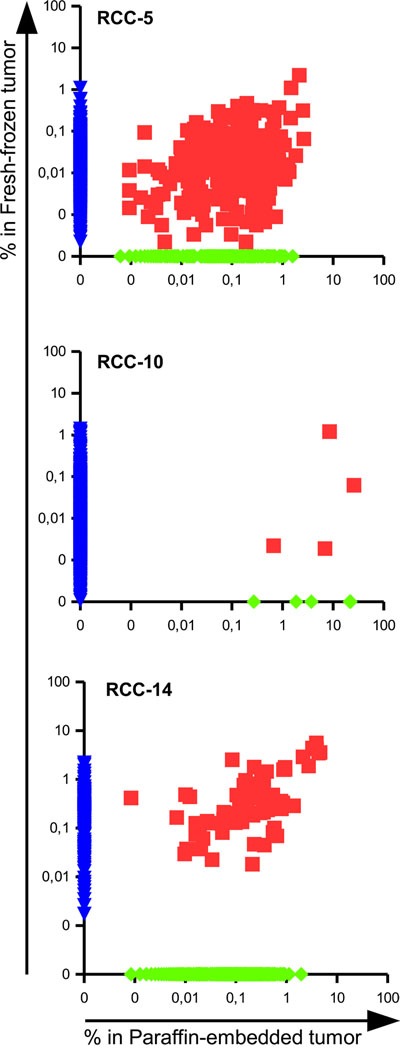
Presence of shared TCRs among different portion of the same tumor reveals a degree of spatial homogeneity in RCC Scatter dot plot representing the frequencies of TCR clonotypes in the paraffin-embedded versus the fresh-frozen tumor tissues of patients RCC-5, RCC-10 and RCC-14. The frequency of not-shared clonotypes was manipulated in order to be displayed on the axis.

Regarding the spatial homogeneity of the infiltrate, a consistent number of shared clonotypes was found within the different patients (Figure 2 and Table 2). Patient RCC-5 had 262 clonotypes shared between the paraffin-embedded and the fresh-frozen tumor tissues representing 61,3 and 15,8% of the respective total TCR repertoires. Among the shared clonotypes, 14 and 13 belong to the top 20 of each sample and three of them were identical, i.e. CATSSGHDYPEAFF was ranked 3^rd^ and 1^st^, CASSQEGSYEKLFF 6^th^ and 2^nd^, while CASSLPGDTEAFF was ranked 19^th^ and 9^th^ in the paraffin-embedded and in the fresh-frozen tumor tissue, respectively.

In patient RCC-10 4 clonotypes were in common among the paraffin-embedded and fresh-frozen tumor tissues. Due to the different number of total reads, the 4 TCR sequences made up 46,6% of the total repertoire in the paraffin-embedded sample, but only 3,66% of the frozen tissue. Interestingly, the shared clonotype CASSSLGTEAFF was the most abundant one of the frozen tissue with a frequency of 3,59%.

In patient RCC-14 the paraffin-embedded and fresh-frozen tumor tissue shared 75 clonotypes that were highly enriched in both samples since they made up to 43,5% of the total TCR repertoire of the paraffin-embedded and 55,5% of the total TCR of the fresh-frozen tumor tissue. Moreover, seven out of the top 10 clonotypes of each sample were in common and already covered 25% of the total repertoire.

Considering the low number of patients (3), sampling replicates (2) and the different processing of the samples, a certain degree of spatial homogeneity could be identified in the three RCC patients suggesting that a single biopsy could provide “consistent” results for the TCR sequencing process as well as being a reliable source for TIL to be expanded *ex vivo* for therapeutic applications.

#### Enrichment of TCR clonotypes in the tumor infiltrate over circulating lymphocytes

**Figure 3:**
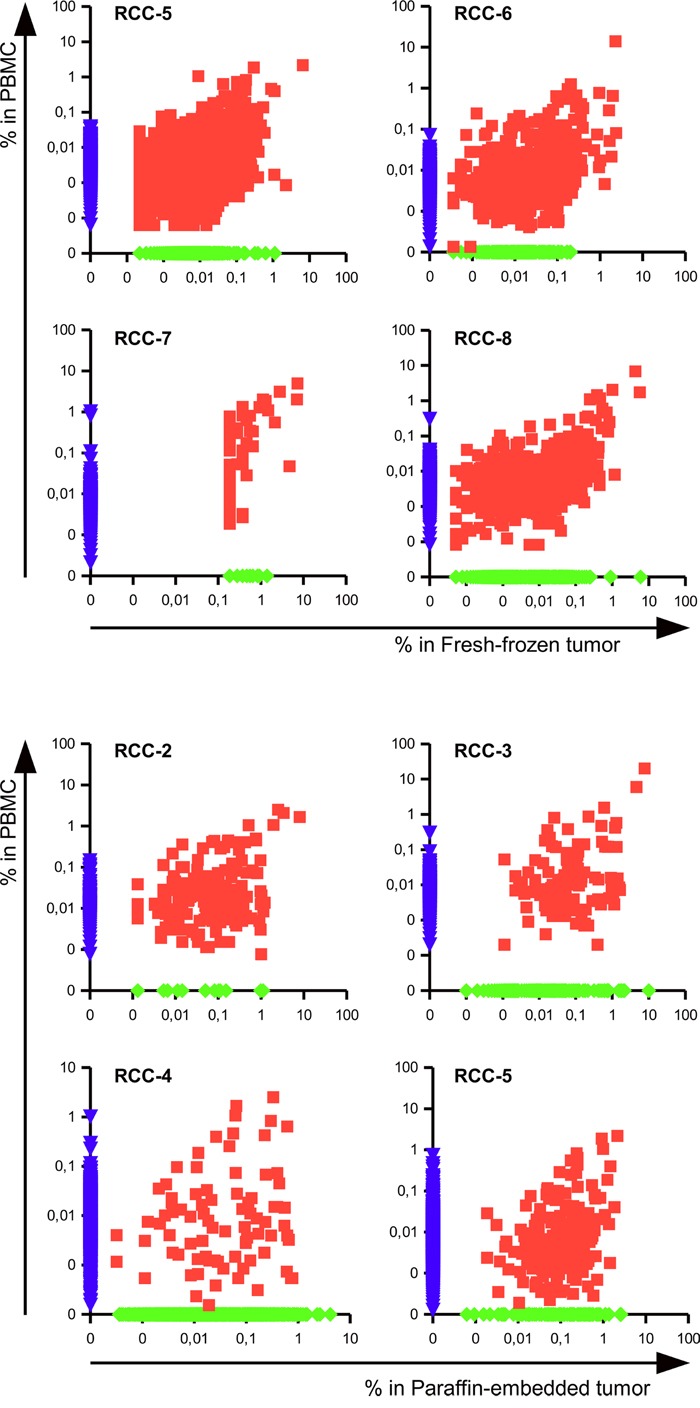
Tumor infiltrating lymphocytes include specific and blood-shared TCR sequences Scatter dot plot representing the frequencies of clonotypes in the PBMC versus the infiltrate of fresh-frozen (patients RCC-5 RCC-6 RCC-7 and RCC-8) or paraffin-embedded tumor tissue (patients RCC-2, RCC-3, RCC-4 and RCC-5). The frequency of not-shared clonotypes was manipulated in order to be displayed on the axis.

**Figure 4:**
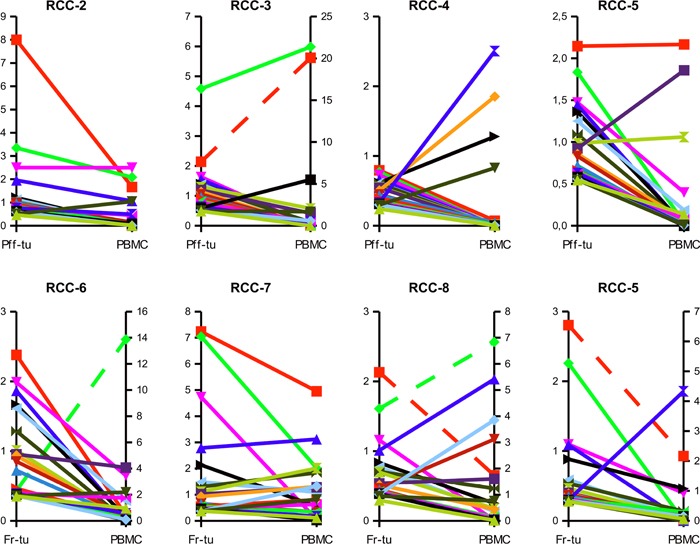
Clonotypes shared between tumor and circulation are frequently enriched within the tumor tissue Frequencies of the top 20 TCR sequences shared between the tumor infiltrate of the paraffin-embedded (Pff-tu) or fresh-frozen (Fr-tu) tumor specimens and the blood of the different RCC patients. Dashed lines report to the secondary Y-axis.

In the seven RCC patients for which it was possible to compare infiltrating versus circulating lymphocytes a high number of shared TCR clonotypes could be identified, as shown in the scattered dot plot of Figure 3. Those clonotypes covered 11,1 to 44,2% of the total TCR repertoire of the tumor, but only 0,44 to 7,12% of the PBMC's suggesting an enrichment of specific clonotypes within the tumor tissues. As shown in Figure 4, evaluation of the top 20 (based on the sum of frequency between the two samples) shared clonotypes indicated in most cases a higher frequency in the tumor infiltrate compared to PBMC. The presence of a different and also opposing “trend” of enrichment of the various clonotypes between the two locations further support the idea of a specific, antigen-driven enrichment / expansion / retention of at least some of the TCR clonotypes in the tumor.

### Comparison among patients

#### Different patterns of TCR clonotypes are shared among RCC patients

**Figure 5:**
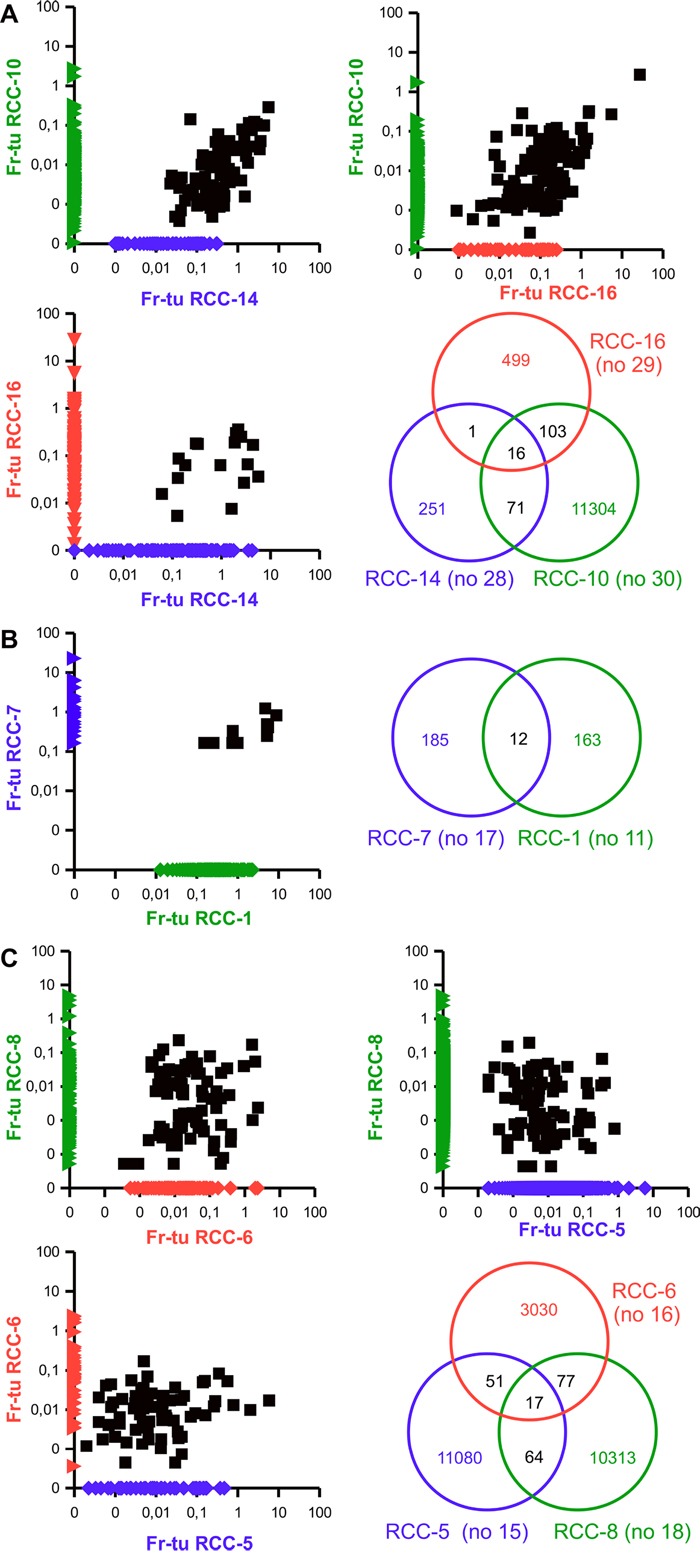
Existence of groups of shared clonotypes among different RCC patients Scattered dot plot representing the frequencies of TCR clonotypes among the fresh-frozen tumor samples of the indicated RCC patients as well as Venn diagram highlighting the number of shared and individual clonotypes. The frequency of non-shared clonotypes was manipulated in order to be displayed on the axis.

Comparison of the clonotypes among the tumor specimens from the different RCC patients highlighted the presence of groups of shared clonotypes (Table 3 and 4, Figure 5).

**Table 3:** CDR3 sequences and frequencies of the clonotypes shared by different RCC patients

	Clonotypes’ CDR3 sequences	RCC-10	RCC-14		RCC-16	nucleotide seq§
		(# 30)	(# 1)	(# 28)	(# 29)	
1	CASSSGTSVYEQYF	0,287	3,902	5,625	0,036	equal
2	CASSPGQGTQPQHF	0,100	3,265	4,405	0,169	equal
3	CASSISGNEQFF	0,025	4,308	3,433	0,066	equal
4	CASSFAPGEQFF	0,108	2,120	2,885	0,027	equal and unique
5	CASSLPPSTDTQYF	0,122	0,084	2,522	0,251	equal and unique
6	CASSLIPPRQGDYGYTF	0,075		2,188	0,357	equal and unique
7	CAAGETQYF	0,029		1,983	0,300	equal
8	CASSSGTGVTASTDTQYF	0,114	2,742	1,872	0,190	equal
9	CASSNTGTDTQYF	0,025	0,937	1,603	0,008	equal
10	CASSSLAGPFLEQFF	0,027	0,156	0,952	0,063	equal
11	CSARETGSIRDDNQPQHF	0,002	0,171	0,332	0,177	equal
12	CASSEFGGTFSDNSPLHF	0,012	0,118	0,300	0,182	unique, equal #28-29-30
13	CASSRDRGSNGYTF	0,012	0,218	0,183	0,063	equal
14	CASSLDRGLGNEQFF	0,010	0,099	0,129	0,034	equal and unique *
15	CASSQDPGLGFSDNQPQHF	0,001	0,016	0,126	0,005	equal and unique
16	CASGATGGHNEQFF	0,000	0,022	0,061	0,016	equal
	Sum of frequency	0,951	18,158	28,599	1,944	
	Total number of unique read	11620		345	633	

§: information about the nucleotide sequence encoding the CDR3 clonotype: equal indicate identical sequence among the various samples; unique indicate that there is only one nucleotide sequence encoding the clonotypes.

*: The clonotype is also present in sample #22, but with a different sequence (see Supplementary Table 2).

**Table 4:** CDR3 sequences and frequencies of the clonotypes shared by different RCC patients

	Clonotypes’ CDR3 sequences	RCC-1 (#11)	RCC-7 (#17)	RCC-5 (#15)	RCC-6 (#16)	RCC-8 (#18)	RCC-9 (#19)	nucleotide seq §
1	CASSDTTSGRNEQFF	9,783	0,928	0,006	0,043	0,049	0,013	E & U
2	CASSLTKGETQYF	5,221	1,391	0,007	0,085	0,014	0,005	E & U
3	CASSPIGPQHF	5,774	0,557	0,001	0,005	3,1E-04	0,001	E & U
4	CAWGQETQYF	5,795	0,278	0,017	0,027	0,035	0,001	E & U
5	CASSTGVSTDTQYF	6,156	0,464	0,016	0,018		0,006	E & U
6	CAWDRGSTDTQYF	0,836	0,371	0,003	0,013	0,117		E
7	CASSPAWDEQFF	1,105	0,186	0,008			0,004	E & U
8	CAWSSGTGGSEQFF	0,850	0,186	0,003		0,017		E & U
9	CAWGRTDYEQYF	0,170	0,186	0,001	0,008			E & U
10	CASSPRGRSYEQYF	0,283	0,186			0,017		E
11	CASTMGGYNYGYTF	1,041	0,186					E & U
12	CASSYLGTGMNTEAFF	0,822	0,186					E & U
13	CASEGPAAGEQYF	0,014			3,6E-04	5,2E-05	1,6E-04	E & U
14	CASSGGTSGLTDTQYF	2,132		0,004	0,025	0,046		E & U
15	CASSSPGYSTYNEQFF		0,186	0,052	0,060	0,007		E
16	CAWSVLGYNEQFF		0,186	0,033	0,044		0,004	E & U
17	CASSQVVFHEQYF			0,387	0,101	0,078	0,014	E & U
18	CASSRPSGRSSSYNEQFF	1,353		0,007	0,026			E & U
19	CASTDLIDSPLHF	1,133		2,2E-04			0,007	E & U
20	CAWSWADYEQYF	0,723			0,015	0,010		E & U
21	CASKVDLNTEAFF	0,326		0,001			0,002	E & U
22	CASTPVKVSGNTIYF		0,649	0,031		0,001		E & U
23	CASSIDPTGDGPQHF		0,186	0,049			0,003	E
24	CASSLQGFDEQFF		0,186	0,016		0,001		E
25	CASSLWRGSTDTQYF		0,186	0,002		0,001		E & U
26	CASSLGGNTEAFF				0,003	0,006	0,535	
27	CASSGTANQPQHF				0,313	0,040	0,003	
28	CASSSQETQYF			0,001	0,207	0,002		
29	CASSLPPSNEQFF			0,181	0,009	1,8E-04		E & U*
30	CASSDLGGGSSYEQYF			0,147	0,016	0,001		E
31	CASSSNYGYTF				0,131	1,3E-04	0,001	
32	CAWKVGGPEGTDTQYF			0,040	0,002	0,046		E* & U
33	CASSQVSAPEAFF			0,005	0,040	0,001		E*& U
34	CASSLVQDPYNEQFF			0,016	0,011	0,011		E & U*
35	CASSSRDSLNYGYTF			0,019	0,009	2,6E-04		E & U
36	CASSTGPPEAQHF			0,011	0,014	0,001		E & U
37	CASSQSTTEAFF			0,007		0,016	0,002	E & U*
38	CASSPELWDLNYEQYF			0,006	0,014	0,001		E & U
39	CASKGQGYNTEAFF			0,014		5,2E-05	0,003	E & U*
40	CASSLMAGLGEQYF			0,009		0,001	0,001	E & U
	Sum of frequencies: - total	43,517	6,684	1,100	1,239	0,520	0,605	
	- core of 12	37,836	5,105	0,062	0,199	0,249	0,030	
	Total unique count	199	176	11212	3175	10471	7679	

§: information about the nucleotide sequence encoding the CDR3 clonotype: E: indicate identical sequence among the various samples; U: indicate that there is only one nucleotide sequence encoding the clonotypes; * indicate that it is true only among the indicated tumor samples but that the clonotypes has other sequences in other samples (see Supplementary Table 3).

Patients RCC-10, RCC-14 and RCC-16 share a core of 16 clonotypes in their fresh-frozen samples covering 0,9, 28,6 and 1,9% of their total TCR repertoire, respectively (Table 3). While only one additional TCR clonotype is shared by patient RCC-14 and patient RCC-16, RCC-10 is sharing additional 71 clonotypes with RCC-14 and 103 with RCC-16 (Figure 5A). Evaluation of the corresponding paraffin-embedded tissue reveals that 14 out of the central core of 16 and 40 of the remaining clonotypes are also present in the paraffin-embedded specimen of RCC-14 (Table 3). Regarding the paraffin-embedded tumor tissue of patients RCC-10, in which only 9 clonotypes were identified, the fifth ranked, belongs to the 71 shared with RCC-14. Evaluation of the nucleotide sequences encoding for these clonotypes highlighted that in the three donors the same sequences encoded the shared clonotype and, in about 30% of the cases it was also the unique sequence expressed by the three donors (Table 2 and Supplementary Table 2). For the remaining clonotypes, at least one patient had multiple nucleotide sequences (up to 7 different) and despite they were frequently derived from the combination of the same V, J and D alleles, there are also multiple cases, in which different V alleles were used and also cases in which different J or different combination of D and J are used. Evaluation of the respective frequencies of the nucleotide sequences coding for the same clonotype within a sample highlighted that in all, but one case the one shared throughout the patients was the most expanded (data not shown). A partial exception to the shared coding sequence is the clonotype CASSEFGGTFSDNSPLHF (12° of Table 3 and Supplementary Table 2), for which the three fresh-frozen samples share an unique sequence, whereas the paraffin-embedded sample utilizes the same V, J and D alleles, but has another unique sequence differing by a single nucleotide mutation.

Patient RCC-1 and RCC-7 share 12 different clonotypes that make up to 37,8 and 5,1% of their total TCR repertoire (Figure 5B and Table 4). Some of these clonotypes are also shared by RCC-5, RCC-6, RCC-8 and / or RCC-9, but at much lower frequencies. Among the tumor infiltrates of these six patients a total of 38 different clonotypes are shared by at least three patients (4 by all six, 2 by five, 8 by four and 24 by three patients; Table 4). Further analysis of these patients highlight a sub-group composed of RCC-5, RCC-6 and RCC-8 that share overall 17 different clonotypes plus other 192 that are shared by only two of them (Figure 5C). Evaluation of the paraffin-embedded tumor tissue from patient RCC-5 reveal overlap only with this second subgroup, with one sequence shared by all and other 15 with the own and one or the other patients’ fresh-frozen tumor tissue (data not shown). Interestingly, whereas all other patients of the group had a carcinoma of the clear cell type, RCC-1 was unclassified and RCC-6 was of chromophobe subtype. Evaluation of the nucleotide sequences of the various clonotypes revealed again that most, but not all the clonotypes shared the same coding sequence among the different samples and that in many cases it was also the unique sequence expressed by the different patients (Table 4 and Supplementary Table 3). As for the other patient group, when multiple sequences were present for the same clonotype, the one shared with the other samples was the most expanded (data not shown). Regarding the usage of the different allele, most of the diversity was due to missing resolution of the allele, with only two clonotypes really using alternative D allele and 5 using multiple V alleles. Exceptions to the sharing “rule” are: the clonotype CASSLGGNTEAFF (26° of Table 4 and Supplementary Table 3), for which the tumors of RCC-6, RCC-8 and RCC-9 (samples #16, #18 and #19) utilizes different V alleles (and also D when defined), whereas the PBMC of RCC-7 and RCC-8 (samples #25 and #26) express the same coding sequence. For clonotype CASSGTANQPQHF and CASSSNYGYTF (27° and 31° of Table 4 and Supplementary Table 3) the tumor of RCC-6 and RCC-8 (samples #16 and #18) but not RCC-9 (#19) share the same nucleotide sequence. Finally, clonotype CASSSQETQYF (28° of Table 4 and Supplementary Table 3) is encoded by many different nucleotide sequences and three of them are shared among different samples: one utilizes the TCRBV05-06 allele and is in common between the tumor of patient RCC-6 and RCC-8 and the PBMC of RCC-6 (sample #16, #18 and #24, respectively); the one using TCRBV11-02 is shared between the tumor and PBMC samples of patient RCC-8 (#18 and #26) and the last utilizes TCRBV07-09 and is in common between the PBMC of RCC-5 and RCC-6 (sample #23 and #24).

Since most of the shared clonotypes used the same V allele, that is encoding the CDR1 and CDR2 portion of the TCR, it can be suggested that they should have the same antigen specificity and thus recognize the same epitope on the same HLA molecule, or at least on HLA-molecules belonging to the same family. The HLA-typing was available only for a subset of patients (Supplementary Table 4) and among the five typed patients of the second group no single allele was shared by all of them even if there were recurrent alleles both for class I (HLA-A02 and HLA-B44) and class II molecules (HLA-DR B1*11 and HLA-DQ B1*03), thus suggesting the possible existence of a CD8^+^ as well as a CD4^+^ T cell-restricted response. Phenotypical and functional evaluation of the infiltrate would be needed to confirm the CD4 and / or CD8 restriction of the clonotypes and, for the former, to evaluate whether they are regulatory T cells (Treg) possibly suppressing the other infiltrating cells or if they are T helper cells that can have a promoting role on the anti-tumor immune response. Of note, a previously identified CD4-restricted TCR specific against a shared RCC antigen was found to have an MHC-unrestricted activity [34], which was due to the direct recognition of soluble TRAIL bound to its receptor DR4 [37].

#### Public clonotypes in patients’ PBMC

Comparison among the PBMC of the different RCC patients highlighted the presence of shared clonotypes, with two sequences common to six patients, 18 to five, 53 to four (Supplementary Table 5) and 308 to three (data not shown), but mostly with frequencies < 0,1%. Most of these sequences were PBMC specific since only 142 out of 381 clonotypes (corresponding to 37% of the sequences) were present also in one to four corresponding tumor samples representing less than 0,5% of their TCR repertoire, suggesting that they were not enriched in the tumor when compared to peripheral blood samples. When evaluating the nucleotide sequences encoding the shared clonotypes a different pattern was found with respect to the tumor samples (Supplementary Table 6) since most of the clonotypes were encoded by different sequences in each of the samples. Only for 16 clonotypes there were shared nucleotide sequences but in five cases these were shared within the different probes of the same donor (clonotypes 5°, 22°, 24°, 28° and 31° of Supplementary Table 5 and 6), with clonotype CASSLGRETQYF (22°) having two different coding sequences differing only for two nucleotides shared by tumor and PBMC samples of RCC-8. Of the remaining clonotypes there is one (CASSSQETQYF, 4° of the Supplementary Table 5 and 6) that has four different shared sequences, even if one is within donor RCC-8 probes; one (CASSYGETQYF; 11°) that has three different shared sequences, with one that is RCC-5 specific, and other four clonotypes that have two different shared sequences (CASSLGQGNTEAFF, CASSLGETQYF, CASSPQGNTEAFF and CASSLGPNTEAFF, 6, 8, 21 and 26°, respectively of the Supplementary Table 5 and 6). Evaluation of the usage of the V, D and J allele among the different sequences encoding the shared clonotypes highlighted usage of different V allele in all, but one clonotype (CASSRTGNTEAFF, 43° of Supplementary Table 5 and 6), whereas the J allele was always conserved, unless the software was not able to identify it. The D allele had also multiple cases, in which diversity was due to unresolved identification but also some cases in which both BD01-01 and BD02-01 were used to encode the clonotypes. The high diversity in the sequence encoding the PBMC shared clonotypes, with different V allele that can influence via different CDR1 and CDR2 region the affinity and specificity of the whole TCR, make even more suggestive that the identity in clonotypes among the tumor samples could be the result of a functional selection, i.e. the recognition and following expansion in response to a tumor-associated, if not tumor-specific antigen.

#### Patient-specific clonotypes

Evaluation of all samples together highlighted many clonotypes that were patient-specific, meaning present in all the samples of one patient, but in none of the others. Those ranged from as few as three TCR for RCC-10, where the paraffin sample only provided 9 unique TCRs, to as many as 740 in RCC-8. Regarding the TCR coverage those patient-specific clonotypes represented a mean ± SE of 24.9% ± 6,3 and 20,9% ± 6,5 in the paraffin-embedded and fresh-frozen tumor sample, respectively, and 13,2% ± 2.8 of the PBMC repertoire. A list of the CDR3 sequences with the highest frequency (i.e. > 1% in at least one sample) together with the overall TCR coverage is provided in Table 5. Evaluation of the nucleotide sequencing coding for the various shared clonotypes highlight that all clonotypes had a common sequence shared by the various samples. In some cases it was the unique existing, while in others additional encoding sequences existed, mostly derived by the combination of the same V, D and J alleles (Supplementary Table 7). Differences were mostly due to unresolved sequences and only in 5 cases there were different V allele and in one case two different D alleles.

**Table 5:** Frequency of the patients’ specific clonotypes

	Patient ID	Pff-tu^a^	Fr-Tu^b^	PBMC	nucleotide seq^§^
	**RCC-2**	**No 2**		**No 20**	
1	CASMGQGHEKLFF	2,503		2,498	equal
2	CSVSRQDTQYF	1,211		0,014	equal and unique
3	CSAPDSSTNEKLFF	1,126		0,008	equal and unique
4	CASTPWGAEAFF	0,511		1,046	equal
5	CSAHTQLTDTQYF	1,025		0,021	equal and unique
6	CASSGNKEKLFF	1,003		0,001	equal and unique
	*Others (133x)*	*19,414*		*7,782*	
	*Total (139)*	*26,793*		*11,370*	
	**RCC-3**	**No 3**		**No 21**	
1	CASGTGIYNEQFF	1,631		0,007	equal
2	CASSLGVRAQETQYF	1,549		0,008	equal and unique
3	CASSSRTREKLFF	0,601		1,541	equal
4	CASIHRAGVGTINTGELFF	1,398		0,015	equal and unique
5	CASSFGGSGGYTF	1,382		0,014	equal and unique
6	CASSPGQSGNIQYF	1,314		0,119	equal
7	CSAFEPPMNTEAFF	1,135		0,003	equal and unique
8	CASNVGVYNEQFF	1,055		0,006	equal
	*Others (94x)*	*11,599*		*3,288*	
	*Total (102)*	*21,664*		*5,001*	
	**RCC-4**	**No 4**		**No 22**	
	*Others (49x)*	*6,508*		*2,121*	
	**RCC-5**	**No 5**	**No 15**	**No 23**	
1	CASSLLYSDTQYF	0,923	0,299	1,857	equal
2	CASSQEGSYEKLFF	1,473	1,096	0,398	equal and unique
3	CSGGQGTPGTEAFF	1,443	0,207	0,002	equal and unique
4	CASSYSSTTEAFF	1,251	0,107	0,186	equal
5	CAWSATVNQPQHF	1,092	0,140	0,026	equal
6	CASTLGAEAFF	0,986	0,009	1,061	equal and unique
	*Others (135x)*	*20,854*	*8,772*	*3,969*	
	*Total (141x)*	*28,022*	*10,630*	*7,5*	
	**RCC-6**		**No 16**	**No 24**	
1	CASSQDLWETQYF		1,862	0,021	equal
2	CASSPANKNIQYF		1,286	0,005	equal
3	CASSEAGEYEQYF		1,009	0,017	equal
	*Others (527x)*		*25,107*	*13,432*	
	*Total (530x)*		*29,264*	*13,475*	
	**RCC-7**		**No 17**	**No 25**	
1	CASTTSRVDQPQHF		7,236	4,954	equal and unique
2	CASSVDVNQPQHF		7,050	2,015	equal
3	CASSITSGAYNEQFF		4,731	0,047	equal and unique
4	CASSDIRGITGELFF		2,134	0,551	equal and unique
5	CASSYSKPTDTQYF		1,484	1,094	equal and unique
6	CATISGSSYNSPLHF		0,371	1,328	equal
7	CASSPPSLNTEAFF		0,928	1,313	equal
8	CAISDGTQTGEQYF		1,020	1,279	equal and unique
	*Others (47x)*		*12,445*	*5,969*	
	*Total (55x)*		*37,399*	*18,550*	
	**RCC-8**		**No 18**	**No 26**	
1	CASSRHPDRALEAFF		4,291	6,838	equal
2	CSVEAGTSVSGELFF		5,682	1,741	equal
3	CATSPGTGMGYTF		1,009	2,031	equal
4	CASSNHDRGGTRSEQYF		0,467	1,170	equal
5	CASSQAARYEQYF		1,156	0,008	equal
6	CASSYSQGWDEQYF		0,244	1,091	equal
	*Others (734x)*		*25,415*	*10,309*	
	*Total (740x)*		*38,264*	*23,19*	
	**RCC-10**	**No 9**	**No 30**		
	CASSRDSPSPLHF	25,995	0,062		equal and unique
	CASSSLGTEAFF	16,976	3,595		equal
	*Others (1x)*	*0,663*	*0,002*		
	*Total (3x)*	*43,634*	*3,659*		
	**RCC-14**	**No 1**	**No 28**		
	CASSQGVNEKLFF	0,940	1,768		equal
	*Others (18x)*	*3,534*	*4,685*		
	*Total (19x)*	*4,474*	*6,453*		

TCR clonotypes with a frequency > 1% in at least one of the samples are individually listed with the respective frequencies. With “others” the remaining (< 1% frequency) patients specific clonotypes are indicated, whereas under “total” all clonotypes are counted. Provided is the number of clonotypes (between brackets) and their added frequency.

^a^: Pff-tu: paraffin-embedded tumor tissue; ^b^: Fresh-frozen tumor tissue

§: information about the nucleotide sequence encoding the CDR3 clonotype: equal indicate identical sequence among the various samples; unique indicate that there is only one nucleotide sequence encoding the clonotypes.

### Comparison with other published TCR sequences

In order to evaluate whether some of the identified clonotypes can have a therapeutic value, available data of published TCR sequences were compared to our sequencing results.

Among published RCC-specific TCR [33, 34, 38–40] we found in our collection of clonotypes only 5 sequences that have already been described [41]. Three of them were detected with low frequency in the blood, but not in corresponding paraffin-embedded tumor tissues of RCC-2 (CASSETSSYEQYF, 0,0038%) and RCC-4 (CASSSTVSYEQYF and CASSGTSSYNEQFF, 0,0090% and 0,0051%) respectively. More interestingly, two clonotypes were found within the tumor infiltrate: CASSGTASYEQYF represented 0,0046% of the fresh-frozen tumor tissue of RCC-10, for which the corresponding PBMC sample was missing, while CASSETDSYEQYF represented 0,0199% of the fresh-frozen tumor tissue of RCC-8 and was not present in the circulating lymphocytes.

Shifting disease, but keeping the organ, two clonotypes of our database have been described in the kidney of patients with systemic lupus erythematosus (SLE) [42]. Whereas the clonotype CASSIGTGSYEQY is present in PBMC, but not the fresh-frozen tumor sample of RCC-7, the fresh-frozen tumor of RCC-8 contained 0,002% of the clonotype CASSRGVYEQY that was described as a CD8^+^ T cell clone present in the kidney biopsy of a SLE patient as well as highly expanded in the blood. The fact that most of the kidney infiltrating cells of this patient had the CD28^null^ phenotype of a memory-effector subset could suggest a specific, possible antigen-driven expansion of this clone during the SLE progression and thus a possible specificity against a kidney antigen that, if expanded in our patient, could lead to adverse autoimmune side effects.

Expanding the search to other antigen specificities, some of our clonotypes have been characterized to be CMV specific [43, 44] with some present only in PBMC samples and two that were also present in the tumor infiltrate: CASSLAPGATNEKLFF was present in the PBMC and tumor of RCC-7 (0,19% in both locations), whereas CASSPSRNTEAFF had a more mixed pattern, since it was present in the tumor infiltrate of RCC-14 (0,13% in both paraffin-embedded and frozen tissue) and RCC-16 (0,09% of the fresh-frozen tumor) for which no corresponding blood sample was available. In contrast, it was absent from the tumor, but present in the corresponding PBMC of RCC-2 and RCC-3 (0,0007 and 0,0005%, respectively). The low frequencies of these virus-specific clonotypes in the tumor infiltrate and the lack of enrichment over matched PBMC support the idea that the other clonotypes that are present in high frequency within the tumor and / or highly enriched with respect to PBMC could be specific for locally expressed and possibly tumor-associated, but not kidney-specific antigens.

## MATERIALS AND METHODS

### Patients’ samples

RCC patients were operated in the Clinic of Urology of the Martin Luther University of Halle-Wittenberg. Upon written informed consent, part of the removed tumor was fresh-frozen in liquid nitrogen and / or embedded in paraffin and further stored in liquid nitrogen till further use. Prior to the surgery blood was drawn and PBMC isolated using standard procedures. Collected lymphocytes were then frozen in 10% DMSO solution and stored in liquid nitrogen till further use.

### DNA extraction and TCR sequencing

DNA from frozen PBMC was extracted using the DNA mini kit from Qiagen following manufacturer's instruction. For tumor samples slides from paraffin-embedded tumor or pieces of the fresh-frozen samples were incubated for 30 min at 65 °C in the extraction solution (0,2 M saccharose, 100 mM Tris, 100 mM NaCl, 50 mM EDTA, 0,5% SDS) and then cooled to 37 °C before treatment with RNase and proteinase K for 18 h at 37 °C. DNA was then purified using phenol-chloroform and chloroform extraction followed by NaCl ethanol precipitation. Five μg DNA/sample were used for TCR sequencing by Adaptive Biotechnologies using the immunoSEQ assay as previously described [45].

### Data analysis and statistic evaluation

The sequencing results were evaluated using the immunoSEQ Analyzer 3.0 software. Since in various cases multiple TCR sequences translated into the same amino acid sequence for the CDR3 region (identified by the immunoSEQ software), we will refer to the translated amino acid sequences as “clonotypes” and to the nucleotide sequences as “reads”.

The Shannon Entropy is a measure of sample diversity (i.e. at higher value correspond a higher number of different sequences) and was calculated by summing the productive frequency times the log (base 2) of the same frequency over all productive rearrangements in the sample. Clonality is calculated by normalizing Entropy by the total number of unique productive rearrangements and subtracting the result from 1 and is used to highlight the presence of clonally expanded sequences.

Comparison among the three samples’ groups was performed with the one-way ANOVA. The samples were considered independent due to the few cases of two / three paired samples from the same patients. A value of p < 0,05 was considered significant and indicated with an asterisk (*).

## CONCLUSION

Overall, TCR sequencing could be performed on tumor samples without prior lymphocyte purification. Not only fresh-frozen, but also paraffin-embedded tumor tissues of more than 7 years can be used, but the latter with a lower success rate and possibly lower output reads. Using this strategy the immunogenicity of RCC was confirmed and antigen specific T lymphocytes identified in the tumor microenvironment. However, CD8 vs CD4 restriction and functional characterization of these antigen-specific T cells will be important for their implementation in immunotherapeutic approaches like adoptive therapy and / or the requirement of checkpoint inhibitors or Treg depleting adjuvant therapy. It is noteworthy that many clonotypes identified were patient-specific and might represent T cells specific for patient-restricted unique neo-antigens important for personalized immunotherapy. Since a considerable number of shared TCR enriched in the tumor infiltrate and common to more than three RCC patients have been characterized, it would be important to identify the corresponding epitopes since such shared antigens might be broadly used as immunotherapeutic targets.

## SUPPLEMENTARY MATERIALS TABLES





## Abbreviations

CAR: chimeric antigen receptor
DC: dendritic cells
IL: interleukin
PBMC: peripheral blood mononuclear cells
RCC: renal cell carcinoma
SLE: systemic lupus erythematosus
TCR: T-cell receptor
TIL: tumor infiltrating lymphocyte
Treg: regulatory T cells.
