# Supplementary material for: Identification of patient-specific and tumor-shared T cell receptor sequences in renal cell carcinoma patients
Source: Oncotarget. 2017 Feb 3;8(13):21212–28. doi: 10.18632/oncotarget.15064 (PMC5400578; doi:10.18632/oncotarget.15064)
Supplement: Supplementary file 1 [file oncotarget-08-21212-s001.pdf]

Identification of patient-specific and tumor-shared T cell receptor sequences in renal cell carcinoma patients

SUPPLEMENTARY TABLES

Supplementary Table 1: Patients' characteristics and samples analyzed

Patient	Sex	Age	Histology	TNM stage			Grading	Sample identifiers [a]		
				T	N	M		Pff-tu[b]	Fr-tu[c]	PBMC
RCC-1	M	57 y	Unclassified	pT2			G2		11	
RCC-2	W	68 y	Clear cell	pT2	pN0	cM0	G3	2	(12)	20
RCC-3	W	66 y	Clear cell	pT1b	pNX	pMX	G2	3	(13)	21
RCC-4	M	77 y	Clear cell	pT3b	pNX	cM1	G2	4	(14)	22
RCC-5	W	66 y	Clear cell	pT1b	pNX	pMX	G2	5	15	23
RCC-6	W	64 y	Chromophobe	pT1			G2		16	24
RCC-7	W	75 y	Clear cell	pT1b	pN0	pMX	G1	(6)	17	25
RCC-8	W	67 y	Clear cell	pT1b	pNX	pM0	G2	(7)	18	26
RCC-9	W	59 y	Clear cell	pT3b	pNX	pMX	G3	(8)	19	(27)
RCC-10	M	47 y	Clear cell	pT1a	pNX	pM0	G2	9	30	
RCC-14	M	56 y	Clear cell	pT1a	pNX	pMX	G2	1	28	
RCC-16	M	67 y	Clear cell	pT3a	pN0	pM0	G2	(10)	29	

[a]: Numbers between brackets identify samples without successful sequencing; [b]: Pff-tu, paraffin-embedded tumor tissue; [c]: Fr-tu, fresh-frozen tumor tissue.

Supplementary Table 2: Information regarding the nucleotide sequences encoding the clonotypes of Table 3

See Supplementary File 1

Supplementary Table 3: Information regarding the nucleotide sequences encoding the clonotypes of Table 4

See Supplementary File 1

Supplementary Table 4: Patients' HLA typing

ID	HLA-A	HLA-B	HLA-C	HLA-DR	HLD-DQ
RCC-4	A02 A23	B13 B27	Cw3 Cw6	B1*07, B1*13 B3+ B4+	B1*02, B1*06
RCC-5	A02 A25	B7 B18	Cw7	B1*15, B5+	B1*06
RCC-6	A01	B14 B44		B1*11 B1*12 B3+	B1*03
RCC-7	A02 A24	B18 B44		B1*01, B1*11, B3+	B1*05, B1*03
RCC-8	A02	B07 B57		B1*07, B1*15, B4+, B5+	B1*03, B1*05
RCC-9	A01A32	B44 B57	Cw5 Cw6	B1*08, B1*11, B3+	B1*04, B1*03

Supplementary Table 5: CDR3 sequences and frequencies of the clonotypes shared between patients' PBMC

See Supplementary File 1

Supplementary Table 6: Information regarding the nucleotide sequences of the clonotypes of Supplementary Table S5

See Supplementary File 1

Supplementary Table 7: Information regarding the nucleotide sequences of the clonotypes of Table 5

See Supplementary File 1
