# Supplementary material for: Identification of patient-specific and tumor-shared T cell receptor sequences in renal cell carcinoma patients
Source: Oncotarget. 2017 Feb 3;8(13):21212–28. doi: 10.18632/oncotarget.15064 (PMC5400578; doi:10.18632/oncotarget.15064)
Supplement: Supplementary file 2 [file oncotarget-08-21212-s002.docx]

Supplementary Table 2. Information regarding the nucleotide sequences encoding the clonotypes of Table 3

|  | CDR3 clonotype | Nucleotide sequences | Used alleles ^a^ | | | Resolved alleles ^b^ | | | Sample # |
| --- | --- | --- | --- | --- | --- | --- | --- | --- | --- |
|  |  |  | V | D | J | V | D | J |  |
| 1 | CASSSGTSVYEQYF | 1 shared & 6 single | 2 (+) | 1 | 1 | BV07-09 | BD02-01 | BJ02-07 | #1 #28 #29 #30 |
|  |  |  |  |  |  | No |  |  | #1 |
|  |  |  |  |  |  | BV07-06 |  |  | #30 |
| 2 | CASSPGQGTQPQHF | 1 shared & 4 single | 1 (+) | 1 | 1 | BV05-06 | BD01-01 | BJ01-05 | #1 #20 #28 #29 #30 |
|  |  |  |  |  |  | No |  |  | #1 |
| 3 | CASSISGNEQFF | 1 shared & 14 single | 4 (+) | 1 | 1 | BV07-09 | BD02-01 | BJ02-01 | #1 #28 #29 #30 |
|  |  |  |  |  |  | No |  |  | #1; # 28 |
|  |  |  |  |  |  | BV05-03 |  |  | #1 |
|  |  |  |  |  |  | BV07-02 |  |  | #16 |
|  |  |  |  |  |  | BV05-08 |  |  | 28 |
| 4 | CASSFAPGEQFF | Unique | 1 | 1 | (+) | BV12-03 / BV12-04 | BD02-01 | no | #1 #28 #29 #30 |
| 5 | CASSLPPSTDTQYF | Unique | 1 | 1 | 1 | BV05-01 | BD02-01 | BJ02-03 | #1 #28 #29 #30 |
| 6 | CASSLIPPRQGDYGYTF | unique | 1 | 1 | 1 | No | BD01-01 | BJ01-02 | #28 #29 #30 |
| 7 | CAAGETQYF | 1 shared & 3 single | 1 | 1 | 2 | BV19-01 | BD02-01 | BJ02-05 | #28 #29 #30 |
|  |  |  |  |  |  |  |  | BJ02-04 | #30 |
| 8 | CASSSGTGVTASTDTQYF | 1 shared & 1 single | 1 | 1 | 1 | BV28-01 | BD01-01 | BJ02-03 | #1 #28 #29 #30 |
|  |  |  |  |  |  |  |  |  | #1 |
| 9 | CASSNTGTDTQYF | 1 shared & 2 single | 1 | 2 | 2 | BV19-01 | BD02-01 | BJ02-03 | #1 #28 #29 #30 |
|  |  |  |  |  |  |  |  |  | # 28 |
|  |  |  |  |  |  |  | BD01-01 | BJ02-05 | #1 |
| 10 | CASSSLAGPFLEQFF | 1 shared & 1 single | 1 | 1 | 1 | BV27-01 | BD02-01 | no | #1 #28 #29 #30 |
|  |  |  |  |  |  |  |  |  | #1 |
| 11 | CSARETGSIRDDNQPQHF | 1 shared & 1 single | 1 | 1 | 1 | BV20-01 | BD01-01 | BJ01-05 | #1 #28 #29 #30 |
|  |  |  |  |  |  |  |  |  | #1 |
| 12 | CASSEFGGTFSDNSPLHF | 1 shared & 1 single | 1 | 1 | 1 | BV02-01 | BD02-01 | BJ01-06 | #28 #29 #30 |
|  |  |  |  |  |  |  |  |  | #1 |
| 13 | CASSRDRGSNGYTF | 1 shared & 1 single | 1 | 1 | 1 | BV28-01 | BD01-01 | BJ01-02 | #1 #28 #29 #30 |
|  |  |  |  |  |  |  |  |  | #1 |
| 14 | CASSLDRGLGNEQFF | Unique (& 1 additional) | 2 | 1 | 1 | BV05-01 | BD01-01 | BJ02-01 | #1 #28 #29 #30 |
|  |  |  |  |  |  | BV18-01 |  |  | #22 |
| 15 | CASSQDPGLGFSDNQPQHF | unique | 1 | 1 | 1 | BV04-02 | no | BJ01-05 | #1 #28 #29 #30 |
| 16 | CASGATGGHNEQFF | 1 shared & 1 single | 1 | 1 | 1 | BV12-03 / BV12-04 | BD02-01 | BJ02-01 | #1 #28 #29 #30 |
|  |  |  |  |  |  |  |  |  | #30 |

^a^: (+) indicate presence of unresolved allele; ^b^: no indicate unresolved allele

Supplementary Table 3. Information regarding the nucleotide sequences encoding the clonotypes of Table 4

|  | CDR3 clonotype | Nucleotide sequences | Used alleles ^a^ | | | Resolved alleles ^b^ | | | Sample # |
| --- | --- | --- | --- | --- | --- | --- | --- | --- | --- |
|  |  |  | V | D | J | V | D | J |  |
| 1 | CASSDTTSGRNEQFF | unique | 1 | 1 | 1 | BV06-01 | BD02-01 | BJ02-01 | #11 #15 #16 #17 #18 #19 |
| 2 | CASSLTKGETQYF | unique | 1 | 1 | 1 | BV11-03 | BD01-01 | BJ02-05 | #11 #15 #16 #17 #18 #19 |
| 3 | CASSPIGPQHF | unique | 1 | 1 | 1 | BV07-06 | BD02-01 | BJ01-05 | #11 #15 #16 #17 #18 #19 |
| 4 | CAWGQETQYF | unique | 1 | 1 | 1 | BV30-01 | no | BJ02-05 | #11 #15 #16 #17 #18 #19 |
| 5 | CASSTGVSTDTQYF | unique | 1 | 1 | 1 | BV07-07 | BD02-01 | BJ02-03 | #11 #15 #17 #16 #19 |
| 6 | CAWDRGSTDTQYF | 1 shared & 1 single | 1 | 1 | 1 | BV30-01 | BD01-01 | BJ02-03 | #11 # 15 #16 #17 #18 |
|  |  |  |  |  |  |  |  |  | #18 |
| 7 | CASSPAWDEQFF | unique | 1 | 1 | 1 | BV19-01 | no | BJ02-01 | #11 #15 #17 #19 |
| 8 | CAWSSGTGGSEQFF | unique | 1 | 1 | (+) | BV30-01 | BD01-01 | no | #11 #15 #17 #18 |
| 9 | CAWGRTDYEQYF | unique | 1 | 1 | 1 | BV30-01 | BD01-01 | BJ02-07 | #11 #15 #16 #17 |
| 10 | CASSPRGRSYEQYF | 1 shared & 1 single | 1 | 1 | 1 | BV18-01 | BD02-01 | BJ02-07 | #11 # 17 # 18 |
|  |  |  |  |  |  |  |  |  | #18 |
| 11 | CASTMGGYNYGYTF | unique | 1 | 1 | 1 | BV07-09 | BD02-01 | BJ01-02 | #11 #17 |
| 12 | CASSYLGTGMNTEAFF | unique | 1 | 1 | 1 | no | BD01-01 | BJ01-01 | #11 #17 |
| 13 | CASEGPAAGEQYF | unique | 1 | 1 | 1 | BV25-01 | no | BJ02-07 | #11 #16 #18 #19 |
| 14 | CASSGGTSGLTDTQYF | unique | 1 | 1 | 1 | BV09-01 | BD02-01 | BJ02-03 | #11 #15 #16 #18 |
| 15 | CASSSPGYSTYNEQFF | 1 shared & 2 single | 1 (+) | 1 (+) | 1 | no | BD02-01 | BJ02-01 | #4 #15 #16 #17 #18 |
|  |  |  |  |  |  | BV07-03 |  |  | #15 |
|  |  |  |  |  |  | no | no |  | #15 |
| 16 | CAWSVLGYNEQFF | unique | 1 | 1 | 1 | BV30-01 | no | BJ02-01 | #4 #15 #16 #17 #19 |
| 17 | CASSQVVFHEQYF | unique | 1 | 1 | 1 | BV14-01 | no | BJ02-07 | #4 #15 #16 #18 #19 #22 |
| 18 | CASSRPSGRSSSYNEQFF | unique | 1 | 1 | 1 | BV28-01 | no | BJ02-01 | #11 #15 #16 |
| 19 | CASTDLIDSPLHF | unique | 1 | 1 | 1 | no | no | BJ01-06 | #11 #15 #19 |
| 20 | CAWSWADYEQYF | unique | 1 | 1 | 1 | BV30-01 | BD01-01 | BJ02-07 | #11 #16 18 |
| 21 | CASKVDLNTEAFF | unique | 1 | 1 | 1 | BV05-05 | BD01-01 | BJ01-01 | #11 #15 #19 |
| 22 | CASTPVKVSGNTIYF | unique | 1 | 1 | 1 | BV07-09 | BD01-01 | BJ01-03 | #15 #17 #18 |
| 23 | CASSIDPTGDGPQHF | 1 shared & 2 single | 1 | 1 (+) | 1 | BV19-01 | no | BJ01-05 | #15 #17 #19 #23 |
|  |  |  |  |  |  |  |  |  | #15 |
|  |  |  |  |  |  |  | BD02-01 |  | #15 |
| 24 | CASSLQGFDEQFF | 1 shared & 1 single | 1 | 1 | 1 | BV07-06 | BD01-01 | BJ02-01 | #15 #17 #18 |
|  |  |  |  |  |  |  |  |  | #18 |
| 25 | CASSLWRGSTDTQYF | unique | 1 | 1 | 1 | BV19-01 | BD01-01 | BJ02-03 | #15 #17 #18 |
| 26 | CASSLGGNTEAFF | 1 shared & 12 single | 10 (+) | 2 (+) | 1 | BV11-02 | no | BJ01-01 | #25 #26 |
|  |  |  |  |  |  | BV05-04 |  |  | #18 |
|  |  |  |  |  |  | BV28-01 |  |  | #19 |
|  |  |  |  |  |  | BV07-08 |  |  | #23 |
|  |  |  |  |  |  | BV07-02 |  |  | #23 |
|  |  |  |  |  |  | BV05-01 |  |  | #25 |
|  |  |  |  |  |  | BV07-03 |  |  | #26 |
|  |  |  |  |  |  | BV12-03 / BV12-04 | BD01-01 |  | #18 |
|  |  |  |  |  |  | BV11-03 |  |  | #25 |
|  |  |  |  |  |  | no |  |  | #29 |
|  |  |  |  |  |  | BV13-01 | BD02-01 |  | #16 |
|  |  |  |  |  |  | BV07-03 |  |  | #23 |
|  |  |  |  |  |  | BV11-03 |  |  | #23 |
| 27 | CASSGTANQPQHF | 1 shared & 2 single | 2 | 1 (+) | 1 | BV19-01 | no | BJ01-05 | #16 #18 #24 |
|  |  |  |  |  |  |  |  |  | #16 |
|  |  |  |  |  |  | BV18-01 | BD02-01 |  | #19 |
| 28 | CASSSQETQYF | 3 shared & 9 single | 9 | 2 (+) | 1 | BV05-06 | no | BJ02-05 | #16 #18 #24 |
|  |  |  |  |  |  | BV11-02 |  |  | #18 #26 |
|  |  |  |  |  |  | BV07-09 |  |  | #23 #24 |
|  |  |  |  |  |  | no |  |  | #15 |
|  |  |  |  |  |  | BV13-01 |  |  | #23 |
|  |  |  |  |  |  | BV05-01 | BD01-01 |  | #24 |
|  |  |  |  |  |  | BV07-08 | BD02-01 |  | #15 |
|  |  |  |  |  |  | BV02-01 |  |  | #20 |
|  |  |  |  |  |  | BV05-04 |  |  | #23 |
|  |  |  |  |  |  | BV06-02 / BV06-03 |  |  | #25 |
| 29 | CASSLPPSNEQFF | 1 shared & 2 single | 3 | 1 | 1 | BV11-03 | BD01-01 | BJ02-01 | #15 #16 #18 #23 #5 |
|  |  |  |  |  |  | BV07-02 |  |  | #23 |
|  |  |  |  |  |  | BV07-09 |  |  | #23 |
| 30 | CASSDLGGGSSYEQYF | 1 shared & 5 single | 1 | 1 (+) | 1 | BV06-01 | BD02-01 | BJ02-07 | #15 #16 #18 #23 |
|  |  |  |  |  |  |  |  |  | #15 |
|  |  |  |  |  |  |  | no |  | #15 |
| 31 | CASSSNYGYTF | 1 shared & 3 single | 4 | 1 (+) | 1 | BV05-06 | no | BJ01-02 | #16 #18 #24 |
|  |  |  |  |  |  | BV28-01 |  |  | #29 |
|  |  |  |  |  |  | BV12-03 / BV12-04 |  |  | #30 |
|  |  |  |  |  |  | BV02-01 | BD02-01 |  | #19 |
| 32 | CAWKVGGPEGTDTQYF | 1 shared & 1 single | 1 | 1 | 1 | BV30-01 | BD02-01 | BJ02-03 | #15 #16 #18 |
|  |  |  |  |  |  |  |  |  | #4 |
| 33 | CASSQVSAPEAFF | 1 shared & 1 single | 1 | 1 | 1 | BV04-02 | BD02-01 | BJ01-01 | #15 #16 #18 |
|  |  |  |  |  |  |  |  |  | #4 |
| 34 | CASSLVQDPYNEQFF | 1 shared & 3 single | 1 | 1 | 1 | BV12-03 / BV12-04 | no | BJ02-01 | #3 #15 #16 #18 #21 |
|  |  |  |  |  |  |  |  |  | #21 |
| 35 | CASSSRDSLNYGYTF | unique | 1 | 1 | 1 | BV05-05 | BD01-01 | BJ01-02 | #4 #15 #16 #18 |
| 36 | CASSTGPPEAQHF | unique | 1 | 1 | 1 | BV07-02 | no | no | #4 #15 #16 #18 |
| 37 | CASSQSTTEAFF | 1 shared & 1 single | 1 | 1 | 1 | BV16-01 | no | BJ01-01 | #4 #15 #18 #19 |
|  |  |  |  |  |  |  |  |  | #4 |
| 38 | CASSPELWDLNYEQYF | unique | 1 | 1 | 1 | no | no | BJ02-07 | #4 #15 #16 #18 |
| 39 | CASKGQGYNTEAFF | 1 shared & 6 single | (+) | 1 | 1 | no | BD01-01 | BJ01-01 | #4 #15 #18 #19 #22 |
|  |  |  |  |  |  |  |  |  | #22 |
| 40 | CASSLMAGLGEQYF | unique | 1 | 1 | 1 | BV07-03 | BD02-01 | BJ02-07 | #15 #18 #19 |

^a^: (+) indicate presence of unresolved allele; ^b^: no indicate unresolved allele

Supplementary Table 5. CDR3 sequences and frequencies of the clonotypes shared between patients´ PBMC

|  | CDR3 clonotype | RCC-2 (#20) | RCC-3 (#21) | RCC-4 (#22) | RCC-5 (#23) | RCC-6 (#24) | RCC-7 (#25) | RCC-8 (#26) | in tumor ^a^ | Shared in PBMC | Shared with tumors |
| --- | --- | --- | --- | --- | --- | --- | --- | --- | --- | --- | --- |
| 1 | CASSLTGGTEAFF |  | 0,011 | 0,000 | 0,002 | 0,004 | 0,004 | 0,003 | 3 |  |  |
| 2 | CASSLGRNTEAFF | 0,002 |  | 0,008 | 0,022 | 0,003 | 0,002 | 0,004 |  |  |  |
| 3 | CASSLYNEQFF |  | 0,002 | 0,014 | 0,003 | 0,002 | 0,006 |  | 2 |  | 1x |
| 4 | CASSSQETQYF | 0,010 |  |  | 0,005 | 0,016 | 0,004 | 0,002 | 3 | 1x | 3x |
| 5 | CASSLRGNQPQHF | 0,001 | 0,001 | 0,001 | 0,039 |  |  | 0,005 | 1 |  | 1x |
| 6 | CASSLGQGNTEAFF |  |  | 0,019 | 0,006 | 0,026 | 0,003 | 0,012 | 2 | 1x | 1x |
| 7 | CASSLGGTDTQYF | 0,001 | 0,013 | 0,001 |  | 0,005 | 0,003 |  | 2 |  |  |
| 8 | CASSLGETQYF | 0,002 | 0,016 | 0,008 | 0,014 | 0,006 |  |  | 1 | 2x |  |
| 9 | CASSLGQNYGYTF | 0,005 |  | 0,006 |  | 0,007 | 0,006 | 0,003 | 1 |  |  |
| 10 | CASSPGYEQYF | 0,013 |  |  | 0,005 | 0,011 | 0,001 | 0,002 | 1 | 1x |  |
| 11 | CASSYGETQYF |  | 0,004 | 0,001 | 0,013 | 0,002 | 0,007 |  | 3 | 1x | 2x |
| 12 | CASSLGSNQPQHF |  | 0,004 | 0,001 | 0,022 | 0,001 |  | 0,005 | 1 |  |  |
| 13 | CASSFSGNTIYF | 0,013 | 0,003 | 0,001 | 0,005 | 0,009 |  |  |  |  |  |
| 14 | CASSLAGTDTQYF |  | 0,006 | 0,004 | 0,010 |  | 0,009 | 0,002 |  |  |  |
| 15 | CASSLAETQYF |  | 0,006 | 0,009 | 0,001 |  | 0,004 | 0,006 | 1 |  |  |
| 16 | CASSLGTTDTQYF | 0,005 |  | 0,003 | 0,000 | 0,001 |  | 0,011 | 1 |  |  |
| 17 | CASSPQGNQPQHF | 0,003 |  |  | 0,006 | 0,005 | 0,004 | 0,002 | 1 |  |  |
| 18 | CASSLTGYEQYF | 0,008 |  | 0,002 | 0,003 | 0,003 | 0,001 |  | 1 |  |  |
| 19 | CASSLAGNTEAFF | 0,001 |  | 0,001 | 0,009 | 0,001 | 0,003 |  |  |  |  |
| 20 | CASSAGTGGETQYF |  | 0,007 | 0,000 | 0,001 |  | 0,005 | 0,001 |  |  |  |
| 21 | CASSPQGNTEAFF | 0,016 |  | 0,014 |  | 0,006 |  | 0,292 | 2 | 1x | 1x |
| 22 | CASSLGRETQYF |  | 0,000 | 0,001 | 0,003 |  |  | 0,111 | 1 |  | 2x |
| 23 | CASSFTDTQYF |  |  |  | 0,003 | 0,021 | 0,001 | 0,011 | 2 |  |  |
| 24 | CASSLSGYEQYF | 0,002 |  | 0,001 | 0,000 |  |  | 0,002 | 2 |  | 1x |
| 25 | CASSLGGETQYF | 0,144 | 0,004 | 0,000 | 0,001 |  |  |  |  |  |  |
| 26 | CASSLGPNTEAFF |  |  | 0,004 | 0,022 |  | 0,013 | 0,006 | 1 | 1x | 1x |
| 27 | CASSLAGGTDTQYF | 0,001 | 0,008 | 0,091 | 0,000 |  |  |  | 1 |  |  |
| 28 | CASSLGVSYEQYF |  |  | 0,013 |  | 0,036 | 0,014 | 0,004 | 3 |  | 1x |
| 29 | CASSSSTDTQYF |  |  |  | 0,001 | 0,009 | 0,006 | 0,013 | 2 |  |  |
| 30 | CASSLGQGPYEQYF | 0,032 |  |  |  | 0,005 | 0,007 | 0,011 | 1 |  |  |
| 31 | CASSLGGQETQYF |  | 0,004 |  | 0,003 | 0,002 |  | 0,002 | 2 |  | 1x |
| 32 | CASSPGQGNSPLHF |  | 0,011 | 0,001 |  |  | 0,002 | 0,012 | 3 |  |  |
| 33 | CASSLDSNQPQHF |  | 0,040 | 0,005 | 0,010 | 0,003 |  |  |  | 1x |  |
| 34 | CASSSGTGYGYTF | 0,002 | 0,008 | 0,001 |  |  | 0,007 |  | 1 |  | 1x |
| 35 | CASSQGNTEAFF | 0,005 |  | 0,011 | 0,019 |  |  | 0,007 | 1 |  |  |
| 36 | CASSLGTGNTEAFF |  |  |  | 0,014 | 0,003 | 0,003 | 0,010 |  |  |  |
| 37 | CASSPSTDTQYF | 0,003 | 0,003 | 0,007 |  |  | 0,017 |  |  |  |  |
| 38 | CASSLDPNTEAFF | 0,002 |  | 0,015 | 0,005 |  | 0,007 |  |  |  |  |
| 39 | CASSLEETQYF |  |  | 0,000 | 0,014 | 0,008 | 0,004 |  | 1 | 1x |  |
| 40 | CASSSTGNTEAFF |  |  | 0,015 | 0,002 | 0,003 |  | 0,005 |  |  |  |
| 41 | CASSLVGTDTQYF | 0,006 |  | 0,007 |  | 0,010 | 0,001 |  |  |  |  |
| 42 | CASSPQGYEQYF | 0,006 |  |  | 0,002 |  | 0,000 | 0,011 | 1 |  |  |
| 43 | CAWSVQGNQPQHF | 0,005 | 0,009 |  | 0,001 |  | 0,008 |  |  |  |  |
| 44 | CASSRTGNTEAFF |  |  | 0,008 | 0,003 |  | 0,001 | 0,007 | 1 |  |  |
| 45 | CASSLSGSGANVLTF | 0,008 | 0,003 |  | 0,008 |  |  | 0,003 |  |  |  |
| 46 | CASSLGQGGTEAFF | 0,001 |  | 0,008 |  | 0,010 | 0,004 |  |  |  |  |
| 47 | CASSLGPDTQYF |  |  |  | 0,001 | 0,006 | 0,010 | 0,006 |  |  |  |
| 48 | CASSLEGNTEAFF | 0,006 | 0,008 |  | 0,002 |  |  | 0,005 | 1 |  |  |
| 49 | CASSLGGSTDTQYF | 0,007 |  |  |  | 0,004 | 0,006 | 0,002 | 2 |  |  |
| 50 | CASSLGGELFF |  |  | 0,006 | 0,003 | 0,003 | 0,008 |  |  |  |  |
| 51 | CASSSQGYEQYF | 0,005 |  | 0,005 |  | 0,003 | 0,002 |  | 1 |  |  |
| 52 | CASSLAGGGNEQFF | 0,005 |  |  | 0,001 |  | 0,004 | 0,008 |  |  |  |
| 53 | CASSLGGNQPQHF | 0,006 | 0,004 | 0,002 |  |  | 0,005 |  |  |  |  |
| 54 | CASSLAGGNTEAFF | 0,005 |  |  | 0,007 | 0,003 |  | 0,003 |  |  |  |
| 55 | CASSSLYEQYF | 0,005 |  | 0,000 |  | 0,007 |  | 0,003 |  |  |  |
| 56 | CASSLQGARTEAFF | 0,008 |  | 0,002 | 0,001 | 0,005 |  |  |  |  |  |
| 57 | CASSLEGYGYTF |  |  | 0,007 | 0,001 |  | 0,002 | 0,006 |  |  |  |
| 58 | CASSLRETQYF | 0,006 | 0,004 | 0,004 |  | 0,001 |  |  |  |  |  |
| 59 | CASSLSNQPQHF |  |  |  | 0,001 | 0,001 | 0,008 | 0,004 |  |  |  |
| 60 | CASSGDSYEQYF |  | 0,009 | 0,002 |  |  | 0,002 | 0,001 |  |  |  |
| 61 | CASSLVNTEAFF |  | 0,007 |  | 0,000 |  | 0,005 | 0,001 |  |  |  |
| 62 | CASSLAYEQYF | 0,001 |  | 0,005 |  | 0,001 | 0,005 |  |  |  |  |
| 63 | CASSLVAGGTGELFF |  |  | 0,006 | 0,003 |  | 0,001 | 0,001 |  |  |  |
| 64 | CASSLGGEQFF |  | 0,003 |  | 0,001 | 0,001 |  | 0,004 | 1 |  |  |
| 65 | CASSLEGSSYEQYF |  | 0,001 |  | 0,001 | 0,003 |  | 0,005 |  |  |  |
| 66 | CASSLDGDSGNTIYF | 0,002 | 0,002 | 0,002 |  | 0,003 |  |  |  |  |  |
| 67 | CASSLYSNQPQHF |  |  | 0,000 | 0,002 |  | 0,002 | 0,002 | 1 |  |  |
| 68 | CASSLRENTEAFF |  |  | 0,001 | 0,002 |  | 0,004 | 0,001 |  |  |  |
| 69 | CASSLQNTEAFF | 0,001 |  | 0,002 |  | 0,002 | 0,002 |  |  |  |  |
| 70 | CASSLGTVNTEAFF |  |  | 0,002 |  | 0,002 | 0,001 | 0,001 |  |  |  |
| 71 | CASSLAGQPQHF |  |  |  | 0,001 | 0,001 | 0,001 | 0,002 |  |  |  |
| 72 | CASSLGGSNQPQHF | 0,002 |  | 0,001 | 0,002 |  | 0,001 |  |  |  |  |
| 73 | CASSRDRGTDTQYF |  |  |  | 0,001 | 0,000 | 0,001 | 0,001 |  |  |  |

^a^: number of tumor samples in which the clonotype is also present

Supplementary Table 6. Information regarding the nucleotide sequences of the clonotypes of supplementary Table 5

|  | CDR3 clonotype | nucleotide seq | V allele ^a^ | D allele ^a^ | J allele ^a^ | Sample # |
| --- | --- | --- | --- | --- | --- | --- |
| 1 | CASSLTGGTEAFF | 10 single | 6 | 2 (+) | 1 | #16; #19; #21; #22; #23; #24; #25; #26; #30 |
| 2 | CASSLGRNTEAFF | 7 single | 6 | 2 (+) | 1 | #20; #22; #23; #24; #25; #26 |
| 3 | CASSLYNEQFF | 1 shared | BV07-09 | no | BJ02-01 | #5 #15 |
|  |  | 20 single | 8 (+) | 1 (+) | BJ02-01 | #5; #15; #21; #22; #23; #24; #25 |
| 4 | CASSSQETQYF | 4 shared | BV06-02 / BV06-03 | BD02-01 | BJ02-05 | #15 #25 |
|  |  |  | BV05-06 | no | BJ02-05 | #16 #18 # 24 |
|  |  |  | BV07-09 | no | BJ02-05 | #23 #24 |
|  |  |  | BV11-02 | no | BJ02-05 | #18 #26 |
|  |  | 7 single | 6 (+) | 2 (+) | BJ02-05 | #15; #20; #23; #24 |
| 5 | CASSLRGNQPQHF | 1 shared | BV27-01 | BD01-01 | BJ01-05 | #18 #26 |
|  |  | 7 single | 4 | 2 (+) | BJ01-05 | #18; #20; #21; #22; #23; #26 |
| 6 | CASSLGQGNTEAFF | 2 shared | BV18-01 | BD01-01 | BJ01-01 | #16 #24 |
|  |  |  | BV07-09 | BD01-01 | BJ01-01 | #22 #26 |
|  |  | 11 single | 7 | BD01-01 | BJ01-01 | #19; #22; #23; #24; #25; #26 |
| 7 | CASSLGGTDTQYF | 7 single | 6 | 1 (+) | 1 | #16; #18; #20; #21; #22; #24; #25 |
| 8 | CASSLGETQYF | 2 shared | BV05-01 | no | BJ02-05 | #22 #24 |
|  |  |  | BV05-06 | no | BJ02-05 | #21 #23 |
|  |  | 12 single | 10 | 1 (+) | BJ02-05 | #5; #20; #21; #23; #24 |
| 9 | CASSLGQNYGYTF | 6 single | 6 | 1 (+) | 1 | #18; #20; #22; #24; #25; #26 |
| 10 | CASSPGYEQYF | 1 shared | BV18-01 | no | BJ02-07 | #23 #24 |
|  |  | 9 single | 6 | 2 (+) | BJ02-07 | #19; #20; #23; #24; #25; #26 |
| 11 | CASSYGETQYF | 3 shared | no | noBJ02-05 | BJ02-05 | #19 #23 |
|  |  |  | BV06-02 / BV06-03 | no | BJ02-05 | #23 #25 |
|  |  |  | BV06-02 / BV06-03 | no | BJ02-05 | #15 #23 |
|  |  | 7 single | 2 (+) | no | BJ02-05 | #15; #21; #22; #24; #30 |
| 12 | CASSLGSNQPQHF | 8 single | 5 (+) | 2 (+) | 1 | #15; #21; #22; #23; #24; #26 |
| 13 | CASSFSGNTIYF | 5 single | 5 | 1 (+) | 1 | #20; #21; #22; #23; #24 |
| 14 | CASSLAGTDTQYF | 5 single | 5 | 2 (+) | 1 | #21; #22; #23; #25; #26 |
| 15 | CASSLAETQYF | 7 single | 5 | 1 (+) | 1 | #19; #21; #22; #23; #25; #26 |
| 16 | CASSLGTTDTQYF | 6 single | 5 | 1 (+) | 1 | #15; #20; #22; #23; #24;#26 |
| 17 | CASSPQGNQPQHF | 7 single | 3 | 1 (+) | 1 | #19; #20; #23; #24; #25; #26 |
| 18 | CASSLTGYEQYF | 6 single | 5 | 2 | 1 | #18; #20; #22; #23; #24; #25 |
| 19 | CASSLAGNTEAFF | 6 single | 4 | 2 (+) | 1 | #20; #22; #23; #24; #25 |
| 20 | CASSAGTGGETQYF | 5 single | 4 | 1 (+) | 1 | #21; #22; #23; #25; #26 |
| 21 | CASSPQGNTEAFF | 2 shared | BV07-09 | BD01-01 | BJ01-01 | #18 #19 #26 |
|  |  |  | BV18-01 | BD01-01 | BJ01-01 | #22 #24 |
|  |  | 6 single | 3 | BD01-01 | BJ01-01 | #18; # 20; #22 |
| 22 | CASSLGRETQYF | 2 shared | BV28-01 | no | BJ02-05 | #18 #26 |
|  |  |  | BV28-01 | BD01-01 | BJ02-05 | #18 #26 |
|  |  | 5 single | 5 | 2 (+) | BJ02-05 | #18; #21; #22; #23 |
| 23 | CASSFTDTQYF | 8 single | 8 | (+) | 1 | #18; #19; #23; #24; #25; #26 |
| 24 | CASSLSGYEQYF | 1 shared | BV05-06 | BD02-01 | BJ02-07 | #5 #15 # 23 |
|  |  | 3 single | 3 | 2 (+) | BJ02-07 | #20; #22; #26 |
| 25 | CASSLGGETQYF | 4 single | 4 | 1 (+) | 1 | #21; #21; #22; #23 |
| 26 | CASSLGPNTEAFF | 2 shared | BV07-03 | BD01-01 | BJ01-01 | #22# 23 |
|  |  |  | BV12-03 / BV12-04 | BD01-01 | BJ01-01 | #15 #23 #25 |
|  |  | 4 single | 3 | no | BJ01-01 | #23; #25; #26 |
| 27 | CASSLAGGTDTQYF | 5 single | 4 | 1 (+) | 1 | #15; #20; #21; #22; #23 |
| 28 | CASSLGVSYEQYF | 1 shared | BV28-01 | no | BJ02-07 | #16 #24 |
|  |  | 7 single | 5 | no | BJ02-07 | #18; #22; #25; #26; #30 |
| 29 | CASSSSTDTQYF | 7 single | 5 (+) | 1 | 1 | #15; #23; #24; #25; #26; #30 |
| 30 | CASSLGQGPYEQYF | 7 single | 5 (+) | 1 | 1 | #15; #20; #24; #25; #26 |
| 31 | CASSLGGQETQYF | 1 shared | BV05-01 | no | BJ02-05 | #16 #24 |
|  |  | 5 single | 4 | 2 (+) | BJ02-05 | #16; #21; #23; #26; #30 |
| 32 | CASSPGQGNSPLHF | 7 single | 5 | 1 | 1 | #5; #15; #19; #21; #22; #25; #26 |
| 33 | CASSLDSNQPQHF | 1 shared | BV05-01 | no | BJ01-05 | #21 #22 #23 |
|  |  | 5 single | 3 | no | BJ01-05 | #21 #23 #24 |
| 34 | CASSSGTGYGYTF | 1 shared | BV07-02 | BD01-01 | BJ01-02 | #25 #29 |
|  |  | 3 single | 2 (+) | BD01-01 | BJ01-02 | #20; #21; #22 |
| 35 | CASSQGNTEAFF | 7 single | 5 | 1 (+) | 1 | #19; #20; #22; #23; #26 |
| 36 | CASSLGTGNTEAFF | 6 single | 4 | 1 (+) | 1 | #23; #24; #25; #26 |
| 37 | CASSPSTDTQYF | 7 single | 6 | 2 (+) | 1 | #20; #21; #22; #25 |
| 38 | CASSLDPNTEAFF | 4 single | 2 | (+) | 1 | #20; #22; #23; #25 |
| 39 | CASSLEETQYF | 1 shared | BV05-01 | no | BJ02-05 | #22 23 |
|  |  | 5 single | 5 | 1 (+) | BJ02-05 | #23; #24; #25; #30 |
| 40 | CASSSTGNTEAFF | 7 single | 5 (+) | 1 | 1 | #22; #23; #24; #26 |
| 41 | CASSLVGTDTQYF | 5 single | 4 (+) | 1 (+) | 1 | #20; #22; #24; #25 |
| 42 | CASSPQGYEQYF | 7 single | 3 (+) | 1 | 1 | #20; #23; #25; #26; #30 |
| 43 | CAWSVQGNQPQHF | 6 single | 1 | 1 | 1 | #20; #21; #23; #25 |
| 44 | CASSRTGNTEAFF | 7 single | 4 (+) | 2 (+) | 1 | #22; #23; #25; #26; #30 |
| 45 | CASSLSGSGANVLTF | 5 single | 5 | 2 | 1 | #20; #21; #23; #26 |
| 46 | CASSLGQGGTEAFF | 4 single | 4 | 1 (+) | 1 | #20; #22; #24; #25 |
| 47 | CASSLGPDTQYF | 4 single | 4 | 1 (+) | 1 | #23; #24; #25; #26 |
| 48 | CASSLEGNTEAFF | 6 single | 5 | 1 (+) | 1 | #19; #20; #21; #23; #26 |
| 49 | CASSLGGSTDTQYF | 7 single | 4 | 2 (+) | 1 | #18; #19; #20; #24; #25; #26 |
| 50 | CASSLGGELFF | 6 single | 4 | (+) | 1 | #22; #23; #24; #25 |
| 51 | CASSSQGYEQYF | 6 single | 5 | 1 | 1 | #15; #20; #22; #24; #25 |
| 52 | CASSLAGGGNEQFF | 4 single | 4 | 2 (+) | 1 | #20; #23; #25; #26 |
| 53 | CASSLGGNQPQHF | 4 single | 4 | 1 (+) | 1 | #20; #21; #22; #25 |
| 54 | CASSLAGGNTEAFF | 5 single | 5 | 2 (+) | 1 | #20; #23; #24; #26 |
| 55 | CASSSLYEQYF | 4 single | 2 (+) | 1 (+) | 1 | #20; #22; #24; #26 |
| 56 | CASSLQGARTEAFF | 4 single | 4 | 1 | 1 | #20; #22; #23; #24 |
| 57 | CASSLEGYGYTF | 4 single | 4 | 1 (+) | 1 | #22; #23; #25; #26 |
| 58 | CASSLRETQYF | 4 single | 2 | 2 | 1 | #20; #21; #22; #24 |
| 59 | CASSLSNQPQHF | 4 single | 4 | 1 (+) | 1 | #23; #24; #25; #26 |
| 60 | CASSGDSYEQYF | 6 single | 3 | 2 (+) | 1 | #21; #22; #25; #26 |
| 61 | CASSLVNTEAFF | 4 single | 4 | 1 (+) | 1 | #21; #23; #25; #26 |
| 62 | CASSLAYEQYF | 4 single | 3 | 1 (+) | 1 | #20; #22; #24; #25 |
| 63 | CASSLVAGGTGELFF | 4 single | 4 | 1 | 1 | #22; #23; #25; #26 |
| 64 | CASSLGGEQFF | 5 single | 5 | (+) | 1 (+) | #18; #21; #23; #24; #26 |
| 65 | CASSLEGSSYEQYF | 4 single | 4 | 1 | 1 | #21; #23; #24; #26 |
| 66 | CASSLDGDSGNTIYF | 4 single | 3 | 1 (+) | 1 | #20; #21; #22; #24 |
| 67 | CASSLYSNQPQHF | 6 single | 4 | 2 | 1 | #19; #22; #23; #25; #26 |
| 68 | CASSLRENTEAFF | 4 single | 4 | (+) | 1 | #22; #23; #25; #26 |
| 69 | CASSLQNTEAFF | 4 single | 3 | 1 (+) | 1 | #20; #22; #24; #25 |
| 70 | CASSLGTVNTEAFF | 4 single | 3 (+) | 1 (+) | 1 | #22; #24; #25; #26 |
| 71 | CASSLAGQPQHF | 6 single | 5 (+) | 1 (+) | 1 | #23; #24; #25; #26 |
| 72 | CASSLGGSNQPQHF | 6 single | 6 | 2 (+) | 1 | #20; #22; #23; #25 |
| 73 | CASSRDRGTDTQYF | 4 single | 4 | 1 | 1 | #23; #24; #25; #26 |

^a^: (+) and no indicate the presence of unresolved allele

Supplementary Table 7. Information regarding the nucleotide sequences of the clonotypes of Table 5

|  | CDR3 clonotype | Nucleotide sequences | Used alleles ^a^ | | | Resolved alleles ^b^ | | | Sample # |
| --- | --- | --- | --- | --- | --- | --- | --- | --- | --- |
|  |  |  | V | D | J | V | D | J |  |
|  | **RCC-2** |  |  |  |  |  |  |  |  |
| 1 | CASMGQGHEKLFF | 1 shared & 2 single | 2 | 1 | 1 | BV05-06 | BD01-01 | BJ01-04 | #2 #20 |
|  |  |  |  |  |  |  |  |  | #20 |
|  |  |  |  |  |  | BV05-04 |  |  | #20 |
| 2 | CSVSRQDTQYF | Unique | 1 | 1 | 1 | BV29-01 | no | BJ02-03 | #2 #20 |
| 3 | CSAPDSSTNEKLFF | Unique | 1 | 1 | 1 | BV20-01 | BD01-01 | BJ01-04 | #2 #20 |
| 4 | CASTPWGAEAFF | 1 shared & 1 single | 1 | 1 | 1 | BV28-01 | BD02-01 | BJ01-01 | #2 #20 |
|  |  |  |  |  |  |  |  |  | #20 |
| 5 | CSAHTQLTDTQYF | Unique | 1 | 1 | 1 | BV20-01 | no | BJ02-03 | #2 #20 |
| 6 | CASSGNKEKLFF | Unique | 1 | 1 | 1 | BV02-01 | BD01-01 | BJ01-04 | #2 #20 |
|  | **RCC-3** |  |  |  |  |  |  |  |  |
| 1 | CASGTGIYNEQFF | 1 shared & 4 single | 1 | 1 | 1 | BV19-01 | BD01-01 | BJ02-01 | #3 #21 |
|  |  |  |  |  |  |  |  |  | #3 |
| 2 | CASSLGVRAQETQYF | Unique | 1 | 1 | 1 | BV05-01 | BD02-01 | BJ02-05 | #3 #21 |
| 3 | CASSSRTREKLFF | 1 shared & 3 single | 1 | 2 | 1 | BV07-08 | BD01-01 | BJ01-04 | #3 #21 |
|  |  |  |  |  |  |  | BD02-01 |  | #21 |
| 4 | CASIHRAGVGTINTGELFF | Unique | 1 | 1 | 1 | no | BD02-01 | BJ02-02 | #3 #21 |
| 5 | CASSFGGSGGYTF | Unique | 1 | 1 | 1 | BV05-01 | no | BJ01-02 | #3 #21 |
| 6 | CASSPGQSGNIQYF | 1 shared & 2 single | 1 | 1 (+) | 1 | BV19-01 | BD01-01 | BJ02-04 | #3 #21 |
|  |  |  |  |  |  |  |  |  | #3 |
|  |  |  |  |  |  |  | no |  | #21 |
| 7 | CSAFEPPMNTEAFF | Unique | 1 | 1 | 1 | BV20-01 | no | BJ01-01 | #3 #21 |
| 8 | CASNVGVYNEQFF | 1 shared & 1 single | 1 | 1 | 1 | BV19-01 | no | BJ02-01 | #3 #21 |
|  |  |  |  |  |  |  |  |  | #3 |
|  | **RCC-5** |  |  |  |  |  |  |  |  |
| 1 | CASSLLYSDTQYF | 1 shared & 7 single | 3 | 1 | 1 | BV06-02 / BV06-03 | BD02-01 | BJ02-03 | #5 #15 #23 |
|  |  |  |  |  |  |  |  |  | # 15 |
|  |  |  |  |  |  |  |  |  | # 23 |
|  |  |  |  |  |  | BV06-04 |  |  | #15 |
|  |  |  |  |  |  | BV10-03 |  |  | #23 |
| 2 | CASSQEGSYEKLFF | Unique | 1 | 1 | 1 | BV04-01 | BD01-01 | BJ01-04 | #5 #15 #23 |
| 3 | CSGGQGTPGTEAFF | Unique | 1 | 1 | 1 | BV29-01 | BD01-01 | BJ01-01 | #5 #15 #23 |
| 4 | CASSYSSTTEAFF | 1 shared & 1 single | (+) | (+) | 1 | no | no | BJ01-01 | #5 #15 #23 |
|  |  |  |  |  |  |  |  |  | #5 |
| 5 | CAWSATVNQPQHF | 1 shared & 2 single | 1 | 1 | 1 | BV30-01 | BD01-01 | BJ01-05 | #5 #15 #23 |
|  |  |  |  |  |  |  |  |  | #15 |
| 6 | CASTLGAEAFF | Unique | 1 | 1 | 1 | BV06-02 / BV06-03 | BD01-01 | BJ01-01 | #5 #15 #23 |
|  | **RCC-6** |  |  |  |  |  |  |  |  |
| 1 | CASSQDLWETQYF | 1 shared & 1 single | 1 | (+) | 1 | BV04-02 / BV04-03 | no | BJ02-05 | #16 #24 |
|  |  |  |  |  |  |  |  |  | #16 |
| 2 | CASSPANKNIQYF | 1 shared & 1 single | 1 | 1 | 1 | BV05-01 | BD02-01 | BJ02-04 | #16 #24 |
|  |  |  |  |  |  |  |  |  | #16 |
| 3 | CASSEAGEYEQYF | 1 shared & 3 single | 1 | (+) | 1 | BV02-01 | no | BJ02-07 | #16 #24 |
|  |  |  |  |  |  |  |  |  | #16 |
|  | **RCC-7** |  |  |  |  |  |  |  |  |
| 1 | CASTTSRVDQPQHF | Unique | 1 | 1 | 1 | BV28-01 | BD01-01 | BJ01-05 | #17 #25 |
| 2 | CASSVDVNQPQHF | 1 shared & 4 single | 4 (+) | (+) | 1 | BV07-02 | no | BJ01-05 | #17 #25 |
|  |  |  |  |  |  | BV07-03 |  |  | #25 |
|  |  |  |  |  |  | no |  |  | #25 |
|  |  |  |  |  |  | BV07-09 |  |  | #25 |
|  |  |  |  |  |  | BV07-06 |  |  | #25 |
| 3 | CASSITSGAYNEQFF | Unique | 1 | 1 | 1 | BV19-01 | BD02-01 | BJ02-01 | #17 #25 |
| 4 | CASSDIRGITGELFF | Unique | 1 | 1 | 1 | BV11-02 | no | BJ02-02 | #17 #25 |
| 5 | CASSYSKPTDTQYF | Unique | 1 | 1 | 1 | BV06-02 / BV06-03 | no | BJ02-03 | #17 #25 |
| 6 | CATISGSSYNSPLHF | 1 shared & 1 single | 2 | (+) | 1 | BV07-08 | no | BJ01-06 | #17 #25 |
|  |  |  |  |  |  | BV07-09 |  |  | #25 |
| 7 | CASSPPSLNTEAFF | 1 shared & 1 single | 1 (+) | 1 (+) | 1 | BV18-01 | BD02-01 | BJ01-01 | #17 #25 |
|  |  |  |  |  |  | no | no |  | #25 |
| 8 | CAISDGTQTGEQYF | Unique | 1 | 1 | 1 | BV10-03 | no | BJ02-07 | #17 #25 |
|  | **RCC-8** |  |  |  |  |  |  |  |  |
| 1 | CASSRHPDRALEAFF | 1 shared & 4 single | (+) | 1 | 1 | no | BD01-01 | BJ01-01 | #18 #26 |
|  |  |  |  |  |  |  |  |  | #18 |
| 2 | CSVEAGTSVSGELFF | 1 shared & 2 single | 1 | 1 | 1 | BV29-01 | BD02-01 | BJ02-02 | #18 #26 |
|  |  |  |  |  |  |  |  |  | #18 |
|  |  |  |  |  |  |  |  |  | #26 |
| 3 | CATSPGTGMGYTF | 1 shared & 1 single | 1 | (+) | 1 | BV24-01 | no | BJ01-02 | #18 #26 |
|  |  |  |  |  |  |  |  |  | #26 |
| 4 | CASSNHDRGGTRSEQYF | 1 shared & 9 single | 1 (+) | 1 (+) | 1 | no | no | BJ02-07 | #18 #26 |
|  |  |  |  |  |  | BV07-03 | no |  | #18 |
|  |  |  |  |  |  | no | BD02-01 |  | #18 |
|  |  |  |  |  |  | no | no |  | #18 |
| 5 | CASSQAARYEQYF | 1 shared & 2 single | 1 | (+) | 1 | BV14-01 | no | BJ02-07 | #18 #26 |
|  |  |  |  |  |  |  |  |  | #18 |
| 6 | CASSYSQGWDEQYF | 1 shared & 4 single | (+) | 1 | 1 | no | BD01-01 | BJ02-07 | #18 #26 |
|  |  |  |  |  |  |  |  |  | #18 |
|  | **RCC-10** |  |  |  |  |  |  |  |  |
| 1 | CASSRDSPSPLHF | Unique | 1 | 1 | 1 | BV02-01 | BD01-01 | BJ01-06 | #9 #30 |
| 2 | CASSSLGTEAFF | 1 shared & 3 single | 2 | (+) | 1 | BV11-02 | no | BJ01-01 | #9 #30 |
|  |  |  |  |  |  |  |  |  | #9 |
|  |  |  |  |  |  |  |  |  | #30 |
|  |  |  |  |  |  | BV07-02 |  |  | #30 |
|  | **RCC-14** |  |  |  |  |  |  |  |  |
| 1 | CASSQGVNEKLFF | 1 shared & 1 single | 1 | 1 | 1 | BV18-01 | BD01-01 | BJ01-04 | #1 #28 |
|  |  |  |  |  |  |  |  |  | #1 |

^a^: (+) indicate presence of unresolved allele; ^b^: no indicate unresolved allele
